# Bioinformatic analyses of mammalian 5'-UTR sequence properties of mRNAs predicts alternative translation initiation sites

**DOI:** 10.1186/1471-2105-9-232

**Published:** 2008-05-08

**Authors:** Jill L Wegrzyn, Thomas M Drudge, Faramarz Valafar, Vivian Hook

**Affiliations:** 1 Computational Science Research Center, San Diego State University, San Diego, USA; 2 Department of Computer Science, San Diego State University, San Diego, USA; 3 Skaggs School of Pharmacy and Pharmaceutical Sciences, University of California, San Diego, La Jolla, USA; 4 Depts. of Pharmacology, Neuroscience, and Medicine, School of Medicine, Uiversity of California, San Diego, La Jolla, USA

## Abstract

**Background:**

Utilization of alternative initiation sites for protein translation directed by non-AUG codons in mammalian mRNAs is observed with increasing frequency. Alternative initiation sites are utilized for the synthesis of important regulatory proteins that control distinct biological functions. It is, therefore, of high significance to define the parameters that allow accurate bioinformatic prediction of alternative translation initiation sites (aTIS). This study has investigated 5'-UTR regions of mRNAs to define consensus sequence properties and structural features that allow identification of alternative initiation sites for protein translation.

**Results:**

Bioinformatic evaluation of 5'-UTR sequences of mammalian mRNAs was conducted for classification and identification of alternative translation initiation sites for a group of mRNA sequences that have been experimentally demonstrated to utilize alternative non-AUG initiation sites for protein translation. These are represented by the codons CUG, GUG, UUG, AUA, and ACG for aTIS. The first phase of this bioinformatic analysis implements a classification tree that evaluated 5'-UTRs for unique consensus sequence features near the initiation codon, characteristics of 5'-UTR nucleotide sequences, and secondary structural features in a decision tree that categorizes mRNAs into those with potential aTIS, and those without. The second phase addresses identification of the aTIS codon and its location. Critical parameters of 5'-UTRs were assessed by an Artificial Neural Network (ANN) for identification of the aTIS codon and its location. ANNs have previously been used for the purpose of AUG start site prediction and are applicable in complex. ANN analyses demonstrated that multiple properties were required for predicting aTIS codons; these properties included unique consensus nucleotide sequences at positions -7 and -6 combined with positions -3 and +4, 5'-UTR length, ORF length, predicted secondary structures, free energy features, upstream AUGs, and G/C ratio. Importantly, combined results of the classification tree and the ANN analyses provided highly accurate bioinformatic predictions of alternative translation initiation sites.

**Conclusion:**

This study has defined the unique properties of 5'-UTR sequences of mRNAs for successful bioinformatic prediction of alternative initiation sites utilized in protein translation. The ability to define aTIS through the described bioinformatic analyses can be of high importance for genomic analyses to provide full predictions of translated mammalian and human gene products required for cellular functions in health and disease.

## Background

Recent investigations indicate that use of alternative initiation codons for protein translation expands the diversity of protein products encoded by mammalian genomes. Alternative initiation addresses the selection of non-AUG codons of mRNAs as start sites for protein translation. Utilization of alternative translation initiation codons (aTIS) has been demonstrated with increasing frequency in mammalian species, as well as in viruses, yeast, and Drosophila [1].

It is known that a single mRNA may give rise to multiple protein isoforms based on utilization of alternative initiation codons [1]. Thus, non-AUG codons, as well as the classical AUG start codon, should be jointly considered in mechanisms for translation of mRNAs.

Numerous mammalian regulatory proteins utilize alternative initiation sites. Many of these proteins utilizing aTIS participate as regulators of metabolism, intracellular signal transduction, transcription and gene expression, growth mechanisms, and related cellular functions. For example, translation of dihydrofolate reductase mRNA can utilize GUG, UUG, CUG, AUA, AUC, and AUU codons as shown in mutagenesis studies [2]. While comprehensive research on all possible mechanisms for selection has not been performed, it has been noted that successful translation at an aTIS can result from leaky scanning or internal entry of ribosomes [1]. Isoforms of the tyrosine kinase *hck *are specified by alternative translation initiation sites [3]. Novel protein isoforms of the Wilms' tumor (WT) suppressor gene, *WTI*, are generated by translation initiation at a CUG codon [4]. Furthermore, human fibroblast growth factor (FGF) utilizes CUG start sites for the production of unique isoforms of FGF [1]. In all three cases, the isoforms result in significant differences in localization and therefore function. Transcription factor, c-myc, is known to result in isoforms from aTIS start site CUG that results in unique transcriptional activity and is expressed higher in high cell density in lymphoid, erythroid and embryo fibroblast cells [9]. These examples represent the expanding group of mammalian proteins produced by alternative initiation for synthesis of proteins that regulate biological processes in health and disease.

While analysis of the human genome utilized AUG for initiation of protein translation from predicted mRNAs [5], alternative initiation sites have not yet been considered for predictions of translated protein gene products. It is essential to computationally define the properties of aTIS to allow prediction of alternative initiation codons and resulting protein products. For this reason, this bioinformatic study analyzed the nucleotide sequence parameters of 5'-UTR regions of mRNAs that utilize aTIS to determine the unique profile of nucleotide sequence properties that can accurately predict alternative initiation codons in mammalian mRNAs.

In the first part of this bioinformatic analysis, a classification tree that assessed the properties of 5'-UTR regions of mRNAs determined the presence or absence of potential aTISs. In the second part of this bioinformatic study, critical parameters of 5'-UTRs were subjected to analyses by an Artificial Neural Network (ANN) that identified the predicted aTIS codon and its location. Systematic evaluation of primary and secondary 5'-UTR regions of mRNA sequences provided knowledge of the most influential parameters for accurate prediction of aTIS. Importantly, training with a group of mRNAs known to utilize aTIS resulted in highly reliable bioinformatic predictions of aTIS by the integrated analyses of the classification tree and the ANN systems. This achievement for bioinformatic prediction of aTIS is an important step towards predicting protein products derived from utilization of alternative translation initiation sites.

## Results

### aTIS Training Set

**Figure 1:**
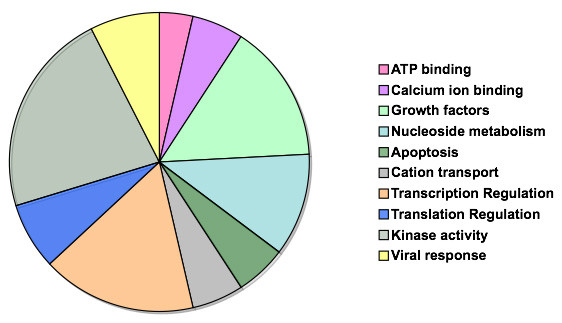
**Functional classification of proteins derived from mRNAs utilizing alternative translation initiation sites**. The positive training set, consisting of verified Mammalian RefSeq mRNA sequences, was analyzed for functional biological categories. These annotations were compiled via BLAST searches and subsequent Gene Ontology (GO) and protein family analysis. The chart depicts the protein functions represented by the identified aTIS sequences. The functions of these proteins are significant for biological regulation.

The classification tree and ANN analyses utilized a training set of mRNA sequences that have been experimentally demonstrated to use alternative initiation sites for protein translation [1,3,7-30]. A positive training set of mammalian mRNAs that utilize aTIS for protein translation was obtained as validated RefSeq sequences (shown in Table 1). This group of 45 non-redundant mRNAs was considered exclusively for training since these mRNAs were validated and confirmed by curators to utilize aTIS [6]. Human, mouse, and rat mRNAs are primarily represented (Table 1). Functional classification of mRNA sequences containing aTIS locations demonstrated that the translated proteins are responsible for highly regulated functions that include growth factors, transcriptional regulation, protein kinases, nucleotide metabolism, viral functions, protein translation, calcium (and cation) and ATP binding, and apoptosis (Fig. 1). It will be important to define the nucleotide sequence properties of the 5'-UTR domains that define use of aTIS for mRNA translation of such regulatory proteins.

**Table 1:** Mammalian mRNAs that Utilize Alternative Initiation Sites (non-AUG) for Protein Translation

**Accession**	**Protein Name**	**Codon**	**Species**	**Location**	**Ref**.
NM_002110	hemopoietic cell kinase (hck)	CUG	Homo sapiens	172	[3]
NM_010407	hemopoietic cell kinase (hck) phosphoribosyl pyrophosphate synthetase 1-like 1	CUG	Mus musculus	186	[3]
NM_175886	(PRPS1L1)	ACG	Homo sapiens	82	[7]
NM_008523	leukocyte tyrosine kinase (ltk), tv 1	CUG	Mus musculus	228	[8]
NM_206941	leukocyte tyrosine kinase (ltk), tv 2	CUG	Mus musculus	228	[8]
NM_002467	v-myc myelocytomatosis viral oncogene homolog (Myc)	CUG	Homo sapiens	525	[9]
NM_012603	myelocytomatosis viral oncogene homolog (Myc)	CUG	Rattus norvegicus	537	[9]
NM_013185	hemopoietic cell kinase (hck)	CUG	Rattus norvegicus	116	[3]
NM_003674	cyclin-dependent kinase 10 (CDK10), tv 1	CUG	Homo sapiens	237	[10]
NM_003674	cyclin-dependent kinase 10 (CDK10), tv 1	CUG	Homo sapiens	255	[10]
NM_052987	cyclin-dependent kinase 10 (CDK10), tv 2	CUG	Homo sapiens	91	[10]
NM_052987	cyclin-dependent kinase 10 (CDK10), tv 2	GUG	Homo sapiens	109	[10]
NM_194444	cyclin-dependent kinase 10 (CDK10), tv 2	CUG	Mus musculus	91	[10]
NM_012890	solute carrier family 30 (zinc), member 2 (Slc30a2)	CUG	Rattus norvegicus	52	[11]
NM_000378	Wilms tumor 1 (WT1), tv A	CUG	Homo sapiens	197	[4]
NM_003213	TEA domain family member 4 (TEAD4), tv 1	UUG	Homo sapiens	275	[12]
NM_013507	p97, repressor translation	GUG	Mus musculus	333	[13]
NM_014293	neuronal pentraxin receptor (NPR)	CUG	Homo sapiens	155	[14]
NM_030689	neuronal pentraxin receptor (NPR)	CUG	Mus musculus	126	[14]
NM_030841	neuronal pentraxin receptor (NPR)	CUG	Rattus norvegicus	115	[14]
NM_009736	Bcl2-associated athanogene 1 (BAG1)	CUG	Mus musculus	34	[15]
NM_004323	BCL2-associated athanogene (BAG1)	CUG	Homo sapiens	73	[16]
NM_003889	Human nuclear receptor (hPAR)	CUG	Homo sapiens	1840	[17]
NM_002006	fibroblast growth factor 2 (FGF2)	CUG	Homo sapiens	303	[18]
NM_002006	fibroblast growth factor 2 (FGF2)	CUG	Homo sapiens	330	[18]
NM_002006	fibroblast growth factor 2 (FGF2)	CUG	Homo sapiens	345	[18]
NM_002006	fibroblast growth factor 2 (FGF2)	CUG	Homo sapiens	69	[18]
NM_021182	minor histocompatability antigen HB-1	CUG	Homo sapiens	108	[19]
NM_130385	tumor suppression (MRVI1)	CUG	Homo sapiens	502	[20]
NM_194464	tumor suppression (MRVI1)	CUG	Mus musculus	647	[20]
NM_003214	TREF-5 transcription enhancer factor	AUA	Homo sapiens	161	[21]
NM_199072	Human I-mfa domain-containing protein (HIC)	GUG	Homo sapiens	264	[22]
NM_031895	calcium channel, voltage-dependent (CACNG8)	CUG	Homo sapiens	104	[23]
NM_023064	spatial stromal protein associated thymii and lymph node	CUG	Mus musculus	84	[24]
NM_020860	stromal interaction molecule 2 (STIM2)	UUG	Homo sapiens	531	[25]
NM_133231	regulatory factor X-associated protein (RFXAP)	ACG	Mus musculus	97	[26]
NM_006386	DEAD box protein (DDX17)	CUG	Homo sapiens	75	[27]
NM_010592	Leucine zipper DNA binding protein (JUND1)	CUG	Mus musculus	139	[28]
NM_138875	Leucine zipper DNA binding protein (JUND1)	CUG	Rattus norvegicus	294	[28]
NM_001025366	vascular endothelial growth factor (VEGF), tv 1	CUG	Homo sapiens	492	[1]
NM_001025367	vascular endothelial growth factor (VEGF), tv 3	CUG	Homo sapiens	666	[1]
NM_001025368	vascular endothelial growth factor (VEGF), tv 4	CUG	Homo sapiens	908	[1]
NM_003376	vascular endothelial growth factor (VEGF), tv 2	CUG	Homo sapiens	645	[1]
NM_001017371	Sp3 transcription factor (SP3), tv 2	AUA	Homo sapiens	385	[29]
NM_001038634	DNase X (LOC515176)	CUG	Bos taurus	185	[30]

This positive set of 45 validated RefSeq mammalian sequences have been identified as containing at least one alternative initiation site. This information was obtained from Genbank records and includes accession, protein name, annotated start codon, species and the relative start position. References for each alternative start site are provided (last column).

### Codon Context

**Figure 2:**
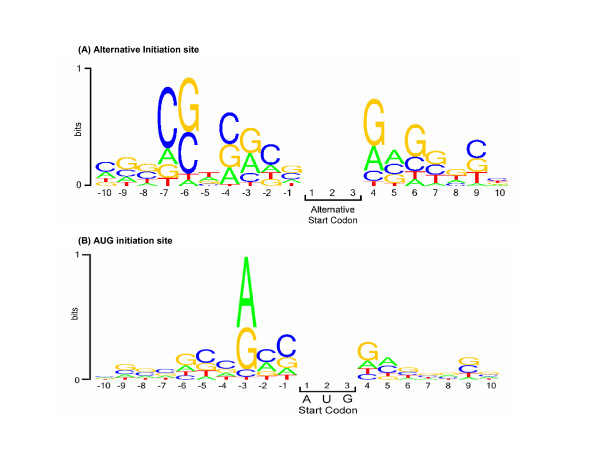
**Unique consensus sequences at aTIS compared to non-aTIS (AUG) in the 5'-UTR region of mRNAs**. Figures 2A and 2B where generated with an adaptation of the WebLogo application [32]. The overall height of the nucleotide stack indicates the sequence conservation at that position, while the height of nucleotide symbols within the stack indicates the relative frequency of each base at that position. The start site is indicated at positions 1, 2, and 3. **(A) Distinct consensus nucleotide sequences near confirmed alternative translation initiation sites in mammalian mRNAs**. The relative abundance of nucleotides (A, T, C, G) at aTISs is shown for a window of -10 to +10 bases at the initiation codon, with the aTIS start codon in positions 1, 2, and 3. Conservation around all of the alternative start sites aTISs is illustrated. Note the strong conservation of (G/C) at the -6 position and C at the -7 position. **(B) Consensus nucleotides near AUG translation initiation sites in mammalian mRNAs**. Graphical representation of relative nucleotide abundances at AUG sites is shown for bases in the region of -10 to +10 bases relative to the initiation codon, with the AUG codon in positions 1, 2, and 3. Conservation at the -3 and +4 locations are consistent with traditional Kozak consensus sequence. These features are distinguished from that of thte aTIS sequences which show conservation at positions -6 and -7 (Figure 2A).

A specific investigation of start codon context was performed in order to determine the most optimal representation for classification and identification. The traditional Kozak consensus sequence ranges from -5 to +6 with the critical base positions located at -3 and +4 [31]. To study the effects of more distant codons, we analyzed a larger window for nucleotides at positions -10 to +10 (Fig. 2). We identified the critical positions in this window that represent unique consensus sequences near aTIS (Fig. 2A) using an adaptation of the WebLogo application [32]. WebLogo presents a graphical representation of the conservation of nucelotides at each position in the supplied window. In order to compare the results of aTIS conservation, we performed the same analysis on the negative set of sequences which used AUG as the start site (Fig. 2B) Notably, the distinct consensus sequences at the aTIS (Fig. 2A) differ from consensus sequences at AUG initiation sites (Fig. 2B). The 5'-UTR of the aTIS sequences showed conservation of G and C in the -6 position, and C in the -7 position, which were present at a significant level in the positive training set. In contrast, the AUG initiation sites showed consensus at position -3 for A and G, and at position 4 for G and A; these characteristics represent features of the Kozak sequence [31]. Clearly, consensus 5'-UTR sequences near aTIS differ from those near AUG initiation sites.

### Sequence Classification

**Figure 3:**
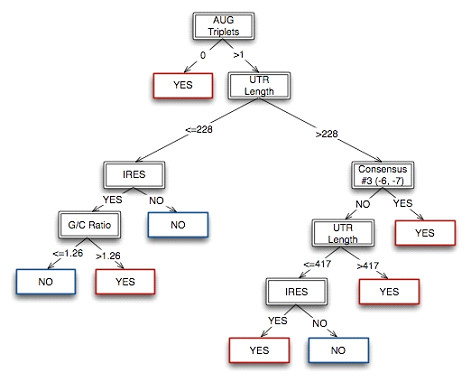
**Classification tree for identification of mRNAs with alternative translation initiation sites (aTIS)**. The C4.5 classification are displayed here in the form of a decision tree. Training, testing, and cross-validation produced a set of 'if-then' rules that allowed the sequences containing the aTISs to be classified independently from sequences that do not possess aTISs. The critical parameters for the classification tree consisted of number of upstream AUGs, 5'-UTR length, consensus sequences (at positions-6/-7), the presence or absence of the Internal Ribosome Entry Site (IRES) structure, as well as G/C ratio.

The goal of the classification tree was to determine the critical parameters that provide the most accurate categorization of sequences that utilize aTIS, and those sequences that do not use aTIS. Analyses of 5'-UTR sequence properties illustrated that mRNAs utilizing aTIS possess distinct parameters with respect to consensus sequences, nucleotide sequence features, and secondary structure (shown in Table 2). The consensus sequence pattern of 5'-UTR regions of mRNAs with aTIS was considered exclusively for consensus at the -6 position (nucleotide G and C) and the -7 position (nucleotide C), in contrast to the traditional Kozak consensus pattern (at positions -3, +4). Nucleotide sequence structural features were also considered with respect to the length of the mRNA, 5'-UTR length, G/C ratio, number of upstream AUGs, and codon bias. Secondary structure was assessed by the presence or absence of Internal Ribosome Entry Site (IRES) which directs translation, and the Glucose Transporter 1 (GLUT1) or Terminal Oligopyrimidine (TOP) features which inhibit translation [33]. These properties allowed classification of the mRNA sequences into the 'Yes' category for the presence of an aTIS, or the 'No' category for the absence of an aTIS (illustrated in Fig. 3).

**Table 2:** 5'-UTR Sequence Parameters Utilized for Analyses by the Classification Tree

**Parameters**	**Description**	**Range**
**Consensus Sequence Patterns**		
Pattern #1 (G/C, C)	Position -6/-7	Yes/No
Pattern #2 (Kozak)	Position -3/+4	Yes/No
**5'-UTR Sequence Parameters**		
5'-UTR Length	Length of 5'-UTR	80 to 2000 bp
mRNA Sequence Length	Full mRNA length	350 to 5000 bp
G/C Ratio	Ratio of G to C	0.4 to 3
GC Percentage	Percentage of GC content	0.3 to 0.9
A/T Ratio	Ratio of A to T	0.2 to 8
Number of AUGs	Number of upstream AUGs from first start site.	0 to 19
Codon Bias	Codonw	0.02 to 1.0
**Secondary Structure**		
IRES	UTRScan/NCBI	Yes/No
GLUT1	UTRScan/NCBI	Yes/No
TOP	UTRScan/NCBI	Yes/No

This table illustrates the parameters (properties) of 5'-UTR regions of mRNAs utilizing aTIS with respect to distinct consensus sequence patterns compared to the traditional Kozak pattern, sequence parameters of 5'-UTR regions, and secondary structure features. The distinct consensus sequence pattern consists of C at position -6 and G/C at position -7 (Fig. 2A). 5'-UTR length is defined as the length of the mRNA sequence from the beginning of the 5'-UTR to the translation initiation site and sequence length as annotated in GenBank. Number of AUGs is determined from the start of the 5'-UTR to the first annotated start site. Secondary structure features considered are Internal Ribosome Entry Sites (IRES), Glucose Transporter 1 (GLUT1), and Terminal Oligopyrimidine (TOP).

For purposes of training the classification tree, 41 of the 45 validated aTIS sequences were used as the positive training set, combined with an equal number (41) from the negative AUG training set randomly selected from the 500 sequences from the negative set; this provided a 'training' set of 82 mRNA sequences. The negative AUG set was composed of validated RefSeq mammalian sequences known to translate with one or more AUG start sites, and which have not been identified as utilizing aTIS [see Additional file 1]. The C4.5 classification tree was used as the algorithm to categorize mRNAs as those that utilize aTIS, and those that do not utilize aTIS. Testing under several conditions defined the properties for the 'Yes' and 'No' decision tree that considered the distinct properties of aTIS with respect to upstream AUGs, 5'-UTR length, distinct consensus sequences at positions -6 and -7, IRES secondary structure, and G/C ratio (Fig. 3).

The classification tree demonstrated a high level of accuracy in classifying mRNAs for aTIS (Table 3). With the 'training' set, the classification tree correctly classified 81 out of 82 sequences. An 'independent testing' set composed of 4 positive validated aTIS sequences and 4 negative AUG sequences, resulted in the correct classification of 7 out of the 8 sequences. In both 'training' and 'independent testing' sets, the classification tree correctly identified all sequences containing aTISs. Due to the small size of the 'training' set, 10-fold 'cross-validation' was performed and resulted in correct classification of 87 sequences out of the total of 90 sequences. Cross-validation is the process by which the data set (consisting of the combined 'training' and 'independent' sets) is divided into 10 random segments and tested for observation of any differences in error resulting from the randomization. The high number of correctly classified aTIS sequences by cross-validation supports decisions of the classification tree. The decision tree then evaluated a 'provisional' aTIS set of sequences. The provisional set contained the positive set of 43 RefSeq sequences *predicted *to contain at least one aTIS combined with a negative set of 43 randomly selected AUG sequences, for a total of 86 sequences in the 'provisional' set. The predicted set had high homology to the validated set, resulting in a total of 10 sequences from the 43 total identified to have conserved aTIS start sites among mammalian species [see Additional file 2]. Testing of the classification tree resulted in correct classification of 83 out of 86 sequences as those that contain aTIS form the 'provisional' set.

The results of testing the various data sets (from Table 3) indicated that the classification tree utilized the 5'-UTR properties consisting of the number of upstream AUG start sites, 5'-UTR length, unique consensus sequence patterns at positions -7 and -6, IRES feature, and G/C ratio. Differences in these properties were observed for 5'-UTR regions of aTIS compared to non-aTIS mRNAs (Table 4). The aTIS sequences were twice as likely to utilize the distinct (-7, -6) consensus pattern, as 60% of aTIS sequences contained this consensus pattern. Also, a higher portion of aTIS-containing sequences possessed IRES secondary structure, compared to non-aTIS sequences. Additionally, aTIS sequences appeared to contain longer 5'-UTR domains compared to non-aTIS sequences. While the G/C ratio was less important than the other variables, it resulted in a split in the classification tree and, therefore, is included as a parameter required for the final classification results. Overall, these properties of the 5'-UTR regions provided predictions of known aTIS within mRNAs by analyses with the classification tree.

**Table 3:** C4.5 Classification Tree Results

**Data Sets**	**# of mRNAs from positive set with aTIS**	**Training Set Size, positive + negative**	**Correctly Classified**	**Incorrectly Classified**	**Mean Absolute Error**	**False Negatives**	**False Positives**
**Training**	41	82	81	1	0.0122	0	1
**Independ. Testing**	4	8	7	1	0.1250	0	1
**Cross-Validation**	45	90	87	3	0.0333	0	3
**Full Negative Set**	0	500	469	31	0.062	0	31
**Provisional**	43	86	83	3	0.0349	0	3

Results of the C4.5 classification tree on training, testing, cross-validation, full negative set and provisional data sets are presented here. The classification tree was able to effectively distinguish between sequences that contain aTIS sites and those that do not. The 45 RefSeq sequences validated as containing alternative start sites were combined with 45 randomly selected sequences without alternative start sites resulting in 90 sequences used for the data sets and training. Of these 90 sequences, 82 were used for the 'training' set and the remaining 8 were used as an 'independent' testing set. 'Cross-validation' of the C4.5 classification tree was performed using 10 fold cross-validation on the full set of 90 sequences, represented by the training and independent testing sets. The 'Full Negative Set' was the full set of 500 non-aTIS sequences which was compiled for generation of testing sets. The resulting performance of this set provides a measure of the ability of the classification tree to generalize to larger datasets. The 'provisional' set consisted of sequences predicted to contain at least one aTIS; this data set performed well during classification. The Mean Absolute Error is calculated as the fraction of incorrectly classified sequences compared to the total number of mRNA sequences in the designated training sets.

**Table 4:** Significant Parameters Utilized by C4.5 Classification Tree as Properties to Determine aTIS

**Parameters**	**aTIS Sequences**		**non-aTIS Sequences**	
	**Mean**	**Std. Dev**.	**Mean**	**Std. Dev**.
**Number of AUGs**	2.41	3.16	1.72	2.73
**5' UTR Length**	477.23	1303.24	100.24	278.97
**(-7,-6) Consensus Sequence**	60%	n/a	27.6%	n/a
**IRES**	58%	n/a	11.84%	n/a
**G/C Ratio**	1.11	0.47	1.00	0.33

Critical parameters in the classification tree are illustrated with respect to their average values for alternative start sites and non-alternative start sites. Differences among these values facilitated the effective classification of mRNAs with aTIS. The number of AUGs is a count of the number of AUGs in the 5'-UTR. Consensus sequences matches against a C in the -7 position from the start site and a G or C in the -6 position. The sequences are also scanned for the IRES secondary structure and the ratio of Guanine and Cytosine in the 5' UTR is also measured. Although the G/C ratio was the least important variable, it resulted in a split in the classification tree and is, therefore, listed as an parameter required for the final classification results.

### aTIS Identification by the Artificial Neural Network (ANN)

**Figure 4:**
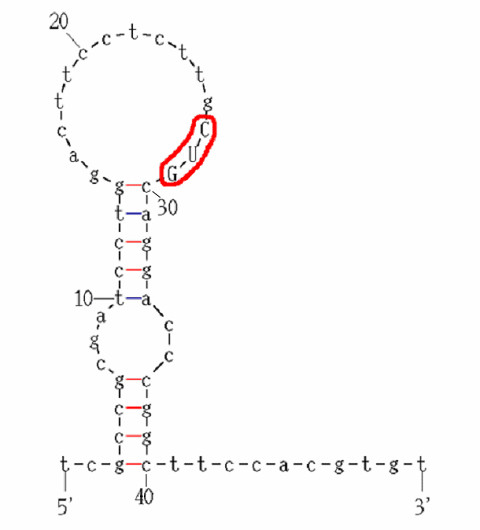
**Secondary structural features of alternative translation initiation sites in 5'-UTR regions of mRNA**. Folding of a representative alternative start site in a stem-loop model using UNAFold centered on the start site is illustrated in this figure. The alternative start site, CUG, is circled in red. Features of this secondary structure served as inputs to the ANN. For each 50 base pair window surrounding the putative alternative initiation site (as shown in this figure), the local stability of the start codon itself and the free energy of the structure were recorded. The window size (50 bp) was experimentally determined as the minimum window size which produced consistent foldings through shifts in the folding window. Based on the scale of 0 to 3 scale, the stability would be measured as a 3 since the codon (all three bases) are present entirely in the loop structure.

**Figure 5:**
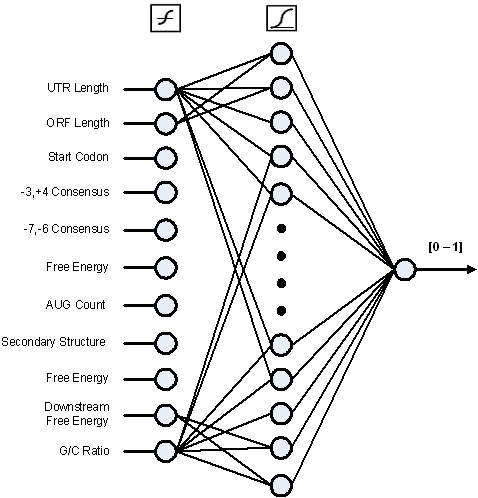
**Organization of artificial neural network (ANN) for identification of alternative translation initiation sites (aTIS)**. To identify aTISs, this study used a feed-forward back-propagation ANN using Matlab's Neural Network toolbox. Artificial Neural Networks are a computational algorithm that uses layers of neurons with weighted edges connecting each layer to perform classification. To determine the specific ANN architecture, this study started with a static training set and modified the number of neurons in the hidden layer of the ANN as well as the activation function used for the neurons in each layer. The resulting ANN contained 10 neurons in the input layer, 20 neurons in the hidden layer and a single output neuron. Inputs to the ANN are normalized in order to negate the effect of measurements in different ranges. The output neuron provides values in the range [0, 1].

Identification of aTISs within mRNAs, based on selected parameters (Table 5), was achieved by analyses with the Artificial Neural Network (ANN) algorithm. Parameters used by the ANN analyses included the two consensus sequence patterns at positions -7 and -6, as well as positions -3 and +4, 5'-UTR length, length of the open reading frame (ORF), number of upstream AUGs, the codon used for translation initiation, and G/C ratio. In addition, secondary structure considerations utilized calculation of the free energy of a 50-base pair stem-loop region containing the initiation codon (Fig. 4) which was presented as input to the ANN. These parameters of 5'-UTR mRNA regions were utilized by the ANN system for analyses and testing for identifying alternative initiation codons (Fig. 5).

The ANN analyses were conducted with three types of data sets (Table 6). Firstly, the 'training' set of the ANN was composed of 41 of the 45 aTIS positive training set, combined with and 41 of the 45 non-aTIS sequences within the same set of mRNAs, for a total of 82 members. The ANN analyses achieved 83% accuracy for predicting aTISs in this 'training' set (Table 6). Of these, the ANN resulted in 7 false negatives and 7 false positives. Secondly, evaluation with a small 'independent testing' set, composed of 4 sequences from the positive aTIS set and 4 randomly selected non-aTIS from the same 4 sequences (total of 8 sequences), resulted in 87.5% accuracy for identifying aTIS. Thirdly, the ANN analyzed a larger 'full' set, consisting of the 45 aTIS sequences from the positive set and 7582 non-aTIS codons obtained from the same positive set of mRNAs, which comprised a total of 7627 sequences for the 'full' set. Analyses of the 'full' set provided a rigorous test of the ANN. The ANN correctly identified 37 of the 45 aTISs, and correctly identified 7087 of the non-aTISs, resulting in a high overall accuracy of 93.4%. Overall, these results demonstrate effective prediction of alternative initiation sites within 5'-UTR regions of mRNAs by the ANN system.

**Table 5:** 5'-UTR Sequence Properties Utilized for Analyses by the Artificial Neural Network (ANN)

**Parameters**	**Description**	**Range**
**Consensus Sequence Patterns**		
Pattern #1 (C/(G/C)	Position -6/-7	Yes/No
Pattern #2 (Kozak)	Position -3/+4	Yes/No
**Consensus Sequence Patterns**		
5'-UTR Length	Length of 5'-UTR	80 to 2000 bp
ORF Length	Length of ORF from the annotated start codon to the stop codon	350 to 5000 bp
Start Codon	Frequency of aTIS in training set	0 to1
Number of AUGs	Number of upstream AUGs from aTIS	0 to 19
G/C Ratio	Normalized ratio of G to C	-1 to 1
**Secondary Structure**		
Free Energy	50 bp UnaFold	-40 to 0
Secondary Structure	UnaFold	0 = stem, 3 = loop

Properties of 5'-UTR sequences of mRNAs using aTIS are shown according to their application in the ANN. The derivation of each feature is shown, as well as the range of representation to the ANN for training and testing. These features are implemented in the ANN analyses which includes refined representations of secondary structure.

**Table 6:** Results of ANN aTIS Identification

**Data Sets**	**# of aTIS sites**	**Data Set Size**	**MSE (All)**	**MSE (aTIS)**	**MSE (non-aTIS)**	**% Correct**	**False Positives**	**False Negatives**
**Training Set**	41	82	0.1165	0.1109	0.1221	82.9	7	7
**Independent Set**	4	8	0.0969	0.1436	0.0502	87.5	0	1
**Full Set**	45	7627	0.0471	0.1138	0.0467	93.4	495	8

Results of the ANN on the training, testing, and full data sets are shown here. For the purpose of identifying the aTISs, the 45 validated alternative start sites were combined with 45 non-alternative start sites randomly selected from the same sequences, resulting in a set of 90 sites. 82 of the 90 sites are used for the 'Training' Set, and the remaining 8 are used as an 'independent testing' set. Further all 7627 potential start sites from the full set of 45 sequences containing alternative start sites were compiled and tested by the ANN. The full set represents all potential start sites present in the 45 positive set of mRNAs, and tests the ability of the ANN to define which sites are likely to act as an alternative initiation start site.

## Discussion

Regulation of protein translation of mRNAs is critical for the control of gene expression. It is important to gain knowledge concerning mechanisms responsible for regulating translation of mRNAs into function protein gene products, especially those not yet discovered. Significantly, increasing numbers of mammalian mRNAs have been found to utilize alternative translation initiation sites (aTIS) for the expression of novel protein isoforms. Therefore, investigation of the parameters that define functional aTISs must be conducted to gain knowledge of factors that participate in generating the diversity of protein gene products.

In this study, bioinformatics analyses of 5'-UTR regions of mRNAs utilizing aTIS by the joint classification tree and ANN system resulted in organization of key properties that provide prediction of aTIS for protein expression. Analyses by the classification tree of 5'-UTR regions of mRNAs utilizing aTIS revealed the importance of distinct consensus sequences (at -7 and -6 positions), upstream AUGs, 5'-UTR sequence length, G/C ratio, and IRES secondary structure as properties that categorized mRNAs as those with and without aTIS. The ANN further analyzed 5'-UTR properties to identity aTIS sites among mRNAs sequences obtained from the classification tree as possessing aTISs. The combined classification tree and ANN analyses for aTIS provide a significant advance towards defining the properties of 5'-UTR sequences for alternative initiation sites. Organization of selected criteria for identification of aTIS by the combined classification tree and ANN algorithm has far reaching potential for elucidation of novel protein isoforms with key regulatory functions.

More specifically, since alternative translation initiation is a rare process in mammalian mRNA, we ensured that the sequence classification algorithm does not exclude any sequences which may contain an aTIS. The classification algorithm is intended to identify sequences which *may *contain aTISs and should be investigated further. The algorithm supplied by the ANN then is able to utilize the results of the sequence classification algorithm and perform analysis on a significantly reduced set of sequences. The computational requirements of secondary structure determination applied in the aTIS location algorithm are significant, so the classification tree acts as a pre-filter.

### Codon Context

The Kozak Consensus sequence as well as secondary structure is known to be critical in the determination of start sites [34,35]. These two parameters were researched in detail for the best representation in the classification and identification algorithms. Independent analysis of the codon context served an important role of identifying how inclusive the window should be as well as the relative importance of individual base positions. In our investigation, we saw a clear preference of the -6 (G/C) and -7 (C) positions within our set of sequences containing aTISs. While the traditional Kozak consensus base positions (-3, +4) were also conserved, they were much more conserved within our negative set of sequences which did not utilize alternative translation. It is clear that within our set, -6 and -7 positions may be necessary if the alternative site is not in an ideal Kozak sequence.

It is also of note, that CUG was the most common aTIS found (80%) within the validated set and resulted in 50% of the aTISs in the predicted set. The GUG start site was the second most common site in the predicted set. This is consistent with initial studies done to compare the relative expression levels of products resulting from single base polymorphisms of the traditional AUG start site [2]. It is also of note, that CUG and GUG are the most common aTIS in species known to use this as form of expression regulation such as Drosophila and E-coli [36,37].

### Secondary Structure

The specific nucleotide composition in a given region aids in determining the stability of the region in terms of the secondary structures that are produced [31,38]. Secondary structures are composed of simple stems and loops resulting from RNA folding and bonding due to chemical interactions of the nucleotide bases. The stability of these structures is a result of the strength of the bonds that form them. Several patterns have been identified that lead to complex secondary structures. These structures have been identified for their specific effects on translation during various biological conditions. The codon's position within the structure effects the efficiency of ribosome scanning and binding [34,39]. The greater the stability, the more difficult it is for the ribosome to bind and begin translation. In terms of complex structures, the individual loops are known to be less stable than the stem portions. The stability is measured in terms of free energy, and energies greater than -50 kcal/mol are known to be inhibitory [33]. While these inhibitory regions aid to halt ribosome scanning and begin translation, stable downstream secondary structures can direct ribosomal scanning and aid in translation initiation [35]. Consideration of the structures as well as the related free energies were critical in examining their effect on alternative initiation.

### Sequence Classification

Since the C4.5 classification algorithm operates on the theory of information gain, parameters yielding the greatest information occur earlier in the classification tree. By using a set of continuous and categorical variables, we achieved a set of 'if-then' rules capable of identifying mRNA sequences which are likely to contain aTISs. The clustering algorithm indicated that the most critical parameters included the number of upstream AUGs, UTR length, the consensus sequence pattern featuring positions -6 and -7, the presence or absence of the IRES, and the G/C ratio.

In situations where no other AUGs are present, alternative initiation may occur at an alternative start site in an ideal consensus sequence. This accounts principally for the fact that a count of zero AUGs is a high indication of alternative initiation in our decision tree. On the other hand, multiple AUGs are often an indication that translation is highly regulated. Unique protein isoforms may be produced at various efficiencies allowing for lower or higher expression levels of a particular product. The length of the 5'-UTR has always been considered an indication of highly regulated sequences. Long 5'-UTRs often contain complex, stable secondary structures, and other long regulatory sequences [33,35,40]. The influence of both the novel consensus sequences (at positions -7 and -6) and the Kozak consensus sequences were consistent with the previous examination of the patterns within our training set. The G/C ratio was also determined to be an influential feature in classification. With a higher the G/C ratio, more inhibition is present for translation. Finally, the presence of secondary structures, specifically the IRES was critical for classification. Notably, IRES structures are important for their directional influence on translation [33].

### aTIS Identification by the Classification Tree and Artificial Neural Network (ANN)

The resulting decision made by the classification tree provided near perfect classification of mRNAs with aTIS, utilizing a defined set of parameters. The purpose of the ANN was to then evaluate these critical parameters to further examine the role of secondary structure in a broader investigation aimed at actual identification of predicted aTIS. By examining all potential start sites with the ANN, this study was able to assess the predictive potential of the resulting network as applied to a much larger data set in which many of the examples were not known to be aTISs. On the initial training set of data, the ANN achieved 82.9% accuracy. When this was expanded to the much larger data set of all potential start sites, the ANN achieved 93.4% overall accuracy in the full data set of all potential start sites using aTIS and AUG start sites. It should be noted that the predicted set was highly homologous to the validated set. Significantly, these results compare favorably with previous prediction algorithms developed for AUG start site location which have been found to yield optimal results from 82% to 94% accuracy [41,42]. We attribute these results to factoring in structural information which was not examined in previous predictive start site algorithms.

We note that our ability to fully consider secondary structure is limited by the inability to determine structures under the biological conditions which have in some cases, modified the folding, to permit the selection of an aTIS. Specific examples of conditions such as stress, heat and salinity effecting the selection of the aTIS and thus resulting isoforms has been noted in fibroblast growth factor (FGF2) where specific conditions favor the AUG isoform with a localization in the cytoplasmic region and the remaining CUG isoforms localize within the nucleus [1].

The ANN system utilized multiple parameters for analyses, consisting of the 5'-UTR length, ORF length, free energy, downstream free energy, local secondary structure, (-3,+4) and (-7,-6) consensus sequences, G/C ratio, aTIS codon, and AUG count. All of these parameters were required for optimum ANN analyses. The parameters involving structural features (free energy, secondary structure, and consensus sequences) were especially important, since their removal resulted in results equivalent to random selection. Significantly, the ANN analyses has defined multiple parameters that together allow identification of alternative initiation sites.

Overall, the classification tree and ANN system provide prediction of alternative initiation sites of mRNAs, based on the distinct profile of 5'-UTR sequence properties of aTIS. The ability to evaluate alternative translation initiation sites in mammalian sequences has far reaching potential for the discovery of previously unknown proteins.

## Conclusion

Alternative initiation of translation is responsible for the synthesis of protein isoforms that have distinct biological functions. To facilitate the discovery of new alternative translation initiation locations, this bioinformatic study provided the combined classification tree-ANN approach for the identification of potential alternative initiation start sites using a critical group of parameters representing sequence properties of 5'-UTR regions of mRNAs. This approach provided a high degree of accuracy when tested against a set of previously identified aTISs. The two-phase approach to this study allows for the classification tree to rapidly identify sequences which may contain aTIS and for the ANN to take on the more computationally intensive process of selecting the start site location. The predictive folding conducted by the ANN is a computationally intensive process which must be repeated for every potential start site used by the ANN. For the purpose of batch processing, the classification tree allows for filtering out sequences which do not contain alternative start sites prior to the ANN phase. Current algorithms for gene prediction in mammalian species do not consider the potential for alternative initiation and as a result, it is likely that this phenomenon has led to an under representation of proteins. Future elucidation of all potential alternative initiation sites for protein translation is required to understand the breadth of genome functions that underlie health and disease conditions.

## Methods

### Data Sets for Classification Tree and ANN (Artificial Neural Network)

The positive aTIS data set used for our experiment was compiled from RefSeq (curated division of GenBank) [6,43]. We obtained a total of 45 non-redundant mammalian mRNAs that have been experimentally demonstrated to use aTIS for protein translation [1,3,6-30,43]. These sequences were manually confirmed to be full-length via Blast against UTRdb, a repository of full length 3'- and 5'-mRNA sequences [44].

An exhaustive search was conducted across several mammalian species in the RefSeq database in order to build a negative data set of non-aTIS sequences. All sequences were required to be validated RefSeq sequences with 5' UTR sequence. From this set, a balanced set was constructed of both highly and non-highly regulated sequences based on the length of the 5' UTR and the length of the ORF. The final set of non-aTIS sequences was comprised of 500 mRNA sequences from a variety of mammalian genomes representing both highly and non-highly regulated genes.

To test the classification tree on new sequences, a set of 43 provisional sequences were compiled. To construct the set of provisional sequences, provisional annotations of aTIS (not experimentally verified) in mRNA sequences of Genbank were compiled. In addition, further provisional aTIS sequences were compiled by analyses of the positive aTIS set within a 120 bp region containing the verified alternative start sites by BLAST searches against the mammalian EST database; resulting sequences that possessed greater than 95% homology with at least one member of the aTIS positive training set were considered provisional aTIS sequences.

Where the clustering algorithm performs on a sequence by sequence basis, the ANN examines individual codons within a specific sequence. In order to build a training set for the ANN, the start site locations in the 45 aTIS positive training sequences were identified. For each instance of an aTIS, a non-aTIS was randomly selected using one of two criteria consisting of (1) a window of -50 to +50 around the aTIS or (2) the closest like codon which was not an aTIS. These two selection criteria helped to ensure that the negative non-aTIS set was as robust as possible in order to avoid trivial classification results.

### Functional Classification

Functional classification was performed on the positive set of aTIS sequences. NCBI's BLAST was used to find homologous sequences to the FASTA formatted input sequences. Gene Ontology (GO) terms were extracted from the top three BLAST hits for each sequence queried. In situations where GO terms were not annotated, domain and protein family annotations were used from BLOCKS, Pfam, and TIGRfam. Annotation rules were employed to assign GO terms to the query sequences. Keyword searches and manual verification allowed for the selection of one GO term in cases where functional discrepancies where identified.

### Parameter Analyses by Classification Tree and ANN

#### General parameters used in analyses by classification tree and ANN

The parameters used for analyses by the classification tree and ANN represent properties of 5'-UTR regions of mRNAs that impact aTIS for alternative translation. This study first assessed global parameters for analysis by the classification tree, and then implemented further parameters for ANN analyses that utilized secondary stem-loop structural information near each potential start site. Extensive research into current theories regarding translation initiation was conducted in order to determine a maximal set of potential parameters to utilize in this study [1,2,31,33-35,38-40,42]. Parameters identified through this research represented primary and secondary structure information as well as content specific information. After identifying an initial set of parameters, further experimentation was conducted to determine optimal parameter representation as well as the minimal set of parameters necessary for sequence classification and aTIS location. Distinct consensus sequence properties near the aTIS was considered with 5'-UTR sequence features that included 5'-UTR length, ORF length, number of upstream AUGs in the 5' UTR region of mRNAs, G/C ratio, and related features. These factors were considered in the classification tree and subsequently in the ANN.

#### Distinct consensus sequences near codons of aTIS for classification tree and ANN

Analyses of consensus nucleotide sequence patterns in each of the positive and negative training sets illustrated distinct properties. These analyses were performed on the positive set of mRNA sequences containing codons for aTIS as CUG, ACG, GUA, UUG, GUG, and AUA. Analyses of the positive training set in a window from -10 to +10 at the alternative start sites identified conservation of cytosine in the -7 position and either a guanine or cytosine in the -6 position. The resulting pattern matched with C(G/C)xxxxxnnnx where the 'nnn' indicates the start site. On the other hand, analyses of the negative training set demonstrated the traditional Kozak Consensus Sequence [31] for a purine in the -3 position and guanine in the +4 position with respect to the AUG initiation site. The resulting pattern matched with (A/G)xxnnnG where the 'nnn' indicates the start site.

#### Codon bias

Different organisms show a variety of preferences for the codons that are used to code amino acids [44-46]. These preferences also vary between different genes within the same organism [45]. More conserved codons for a given organism may aid in translational accuracy and control [46]. Codon bias is a measure of the conservation of codons within a given gene [47]. This study, therefore, utilized the Effective Number of Codons as computed by the "codonw" program [48]. The effective number of codons is a measure of the synonymous codon usage within a given gene [49]. This number was computed for the full length sequence as input to the classification tree.

#### Secondary structure features

For clustering purposes by the classification tree, we used categorical variables (presence or absence) to describe specific secondary structures known to direct or inhibit translation. The 5' UTR sequences of both the positive and negative sets were searched against UTRsite, a database containing the nucleotide patterns of the structures under examination [44]. The results of these queries determined the exact locations of specific structures within these training sets, which included IRE, IRES, GLUT1, and TOP secondary structures. Furthermore, IRESite was used to manually annotate IREs structures that have been experimentally determined, but are not specifically annotated in GenBank [48]. A total of three structures were chosen for investigation by the classification tree consisting of internal ribosome entry site (IRES) [39], as well as Terminal Oligopyrimidine Tract (TOP) and Glucose transporter type-1 (GLUT1) [33].

For the training of the ANN, a more rigorous examination of secondary structure was conducted. Parameters used for representation of structural information included free energy, predicted structure, and local nucleotide composition. Local nucleotide composition utilized the two consensus patterns as well as the G/C ratio around the potential start site. The G/C ratio was measured in a range of [-1, 1]. The range for G/C ratio results in a '-1' if the region is entirely composed of cytosine and '1' if the region is entirely composed of guanine. High values in these categories are indicative of inhibitory patterns [1,31,35].

For ANN analyses, to predict the secondary structure and free energy at alternative start sites, UNAFold was used on a window of 50 nucleotides centered on the start codon. The program uses the melting energies of the bonds between nucleotides to determine the most stable structures for a given sequence [50,51]. UNAFold returns a set of possible structures, ranked in order by the free energy of the structure, from which we considered the first and most stable structure reported. The window size was experimentally determined as the minimum window size which produced consistent foldings through shifts in the folding window. From the output of UNAFold, we obtain two parameters for training the ANN consisting of the free energy of the 50 nucleotide window, as well as a count of the number of nucleotides from the start site which occur in a loop structure. Prediction of secondary structure in a given location is difficult to predict accurately and can easily shift under slight fluctuations of temperature and salinity. To accommodate this uncertainty, the free energy provides a more general measure of structural stability of the region.

### Clustering of Sequences for aTIS by the Classification Tree

The C4.5 implementation of a classification tree in Weka 3.4.1 [52] was used for training using two pre-defined clusters. Class A consisted of sequences that utilize alternative initiation to mediate translation. Class B consists of sequences that are restricted to AUG start sites. The first seven input parameters related to primary sequence features were inputted as discrete values; these parameters consisted of 5'-UTR length, mRNA sequence length, G/C Ratio, GC percentage, A/T ratio, number of AUGs and codon bias. Training was performed through three runs utilizing a random selection of 41 negative instances from the set of 500 non-aTIS sequences compiled from RefSeq resulting in three independent classification trees. In all three cases, the classification trees used the same parameters at each split, with the only variation being in the split value. In order to merge the trees together, the split values were averaged together, resulting in a new tree. The performance of the resulting averaged tree was compared to the previous three and found to be better or equal to the previous three. With the 98.89% accuracy provided by the combined classifcation tree, we deteremined that the results were sufficient to proclude further testing. An independent testing set of four positive and four negative instances was compiled and tested against the resulting classification tree. The four positive instances in the testing set came from the unused aTIS sequences from the full set of 45 aTIS sequences. The four negative instances were randomly selected from the negative set of 500 prior to each run. A 10-fold cross-validation was subsequently performed by dividing the learning sample into ten roughly equal parts, each containing similar distribution for the classification variable.

### Training Artificial Neural Network (ANN) for aTIS Identification

Artificial Neural Networks have been successfully used in the automated location of AUG translation initiation sites [41,53]. In order to locate aTISs, this study used a fully connected feed-forward back-propagation ANN using the Matlab's Neural Network toolbox. Artificial Neural Networks are a computational algorithm which uses layers of neurons with weighted edges connecting each layer to perform classification. The input parameters are passed into the initial layer and the output layer determines the classification results. Using a pre-classified set of data, the ANN is trained to yield correct classification results through a process of updating the weighted edges using back-propagation of the classification error. The process of training the ANN is an iterative process that involves modifying parameter representation, factoring in new parameters and modifying the network structure until the desired performance is obtained.

To determine the specific ANN architecture we used a static training set and modified the number of neurons in the hidden layer of the ANN as well as the activation function used for the neurons in each layer. The resulting ANN contained 10 neurons in the input layer, 20 neurons in the hidden layer and a single output neuron. Inputs to the ANN are normalized in order to negate the effect of measurements in different ranges. The output neuron provides values in the range [0, 1] and is intended to provide a regression value to indicate a probability that a given location is an aTIS.

Parameter selection for the ANN was based on the results from the classification tree, with additional structural parameters factored in. These parameters were modified from the representation used by the classification tree to represent values in reference to the aTIS location. For example, where the sequence classification algorithm measured 5'-UTR length from the beginning of the sequence to initial start site, the aTIS algorithm measures to the aTIS location.

## List of Abbreviations

aTIS: Alternative Translation Initiation Site; ANN: Artificial Neural Network; ORFs: Open Reading Frames; IRES: Internal Ribosome Entry Site; GLUT1: Glucose Transporter type-1; TOP: Terminal Oligopyrimidine.

## Authors' contributions

JLW and TMD participated equally in the specific design, informatics, and analysis. The project was conceived from ongoing studies of the laboratory of VH; bioinformatic analysis for this project was coordinated by FV and VH. All authors contributed to the manuscript preparation, and approved the final manuscript.

## Supplementary Material

Additional file 1 Negative set of Mammalian AUG, non-ATIS, mRNA Sequences. A set of 500 sequences were selected from Mammalian species that translate with one or more AUG start sites. These sequences were selected from the validated RefSeq database. Click here for file

Additional file 2 Provisional aTIS RefSeq mRNA Sequences. The provisional set of sequences were selected both from the annotated provisional non-AUG start sites and conserved non-AUG start sites found in aligned 120 bp fragments containing the alternative start sites from the positive aTIS training set. Click here for file

## References

[B1] Stephanie TourielCB, Sophie Bonnal, Sylvie Audiger, Herve Prats, Anna-Catherine Prats, Stephan Vagner (2003). Generation of Protein Isoform Diversity by Alternative Initiation of Translation at non-AUG Codons. Biology of the Cell.

[B2] Peabody DS (1989). Translation Initiation at non-AUG triplets in Mammalian Cells. Journal of Biological Chemistry.

[B3] Lock P, Ralph S, Stanley E, Boulet I, Ramsay R, Dunn AR (1991). Two isoforms of murine hck, generated by utilization of alternative translational initiation codons, exhibit different patterns of subcellular localization. Mol Cell Biol.

[B4] Bruening W, Pelletier J (1996). A non-AUG translational initiation event generates novel WT1 isoforms. Journal of Biological Chemistry.

[B5] Lander ES, Linton LM, Birren B, Nusbaum C, Zody MC, Baldwin J, Devon K, Dewar K, Doyle M, FitzHugh W, Funke R, Gage D, Harris K, Heaford A, Howland J, Kann L, Lehoczky J, LeVine R, McEwan P, McKernan K, Meldrim J, Mesirov JP, Miranda C, Morris W, Naylor J, Raymond C, Rosetti M, Santos R, Sheridan A, Sougnez C, Stange-Thomann N, Stojanovic N, Subramanian A, Wyman D, Rogers J, Sulston J, Ainscough R, Beck S, Bentley D, Burton J, Clee C, Carter N, Coulson A, Deadman R, Deloukas P, Dunham A, Dunham I, Durbin R, French L, Grafham D, Gregory S, Hubbard T, Humphray S, Hunt A, Jones M, Lloyd C, McMurray A, Matthews L, Mercer S, Milne S, Mullikin JC, Mungall A, Plumb R, Ross M, Shownkeen R, Sims S, Waterston RH, Wilson RK, Hillier LW, McPherson JD, Marra MA, Mardis ER, Fulton LA, Chinwalla AT, Pepin KH, Gish WR, Chissoe SL, Wendl MC, Delehaunty KD, Miner TL, Delehaunty A, Kramer JB, Cook LL, Fulton RS, Johnson DL, Minx PJ, Clifton SW, Hawkins T, Branscomb E, Predki P, Richardson P, Wenning S, Slezak T, Doggett N, Cheng JF, Olsen A, Lucas S, Elkin C, Uberbacher E, Frazier M, Gibbs RA, Muzny DM, Scherer SE, Bouck JB, Sodergren EJ, Worley KC, Rives CM, Gorrell JH, Metzker ML, Naylor SL, Kucherlapati RS, Nelson DL, Weinstock GM, Sakaki Y, Fujiyama A, Hattori M, Yada T, Toyoda A, Itoh T, Kawagoe C, Watanabe H, Totoki Y, Taylor T, Weissenbach J, Heilig R, Saurin W, Artiguenave F, Brottier P, Bruls T, Pelletier E, Robert C, Wincker P, Smith DR, Doucette-Stamm L, Rubenfield M, Weinstock K, Lee HM, Dubois J, Rosenthal A, Platzer M, Nyakatura G, Taudien S, Rump A, Yang H, Yu J, Wang J, Huang G, Gu J, Hood L, Rowen L, Madan A, Qin S, Davis RW, Federspiel NA, Abola AP, Proctor MJ, Myers RM, Schmutz J, Dickson M, Grimwood J, Cox DR, Olson MV, Kaul R, Raymond C, Shimizu N, Kawasaki K, Minoshima S, Evans GA, Athanasiou M, Schultz R, Roe BA, Chen F, Pan H, Ramser J, Lehrach H, Reinhardt R, McCombie WR, de la Bastide M, Dedhia N, Blocker H, Hornischer K, Nordsiek G, Agarwala R, Aravind L, Bailey JA, Bateman A, Batzoglou S, Birney E, Bork P, Brown DG, Burge CB, Cerutti L, Chen HC, Church D, Clamp M, Copley RR, Doerks T, Eddy SR, Eichler EE, Furey TS, Galagan J, Gilbert JG, Harmon C, Hayashizaki Y, Haussler D, Hermjakob H, Hokamp K, Jang W, Johnson LS, Jones TA, Kasif S, Kaspryzk A, Kennedy S, Kent WJ, Kitts P, Koonin EV, Korf I, Kulp D, Lancet D, Lowe TM, McLysaght A, Mikkelsen T, Moran JV, Mulder N, Pollara VJ, Ponting CP, Schuler G, Schultz J, Slater G, Smit AF, Stupka E, Szustakowski J, Thierry-Mieg D, Thierry-Mieg J, Wagner L, Wallis J, Wheeler R, Williams A, Wolf YI, Wolfe KH, Yang SP, Yeh RF, Collins F, Guyer MS, Peterson J, Felsenfeld A, Wetterstrand KA, Patrinos A, Morgan MJ, de Jong P, Catanese JJ, Osoegawa K, Shizuya H, Choi S, Chen YJ (2001). International Human Genome Sequencing Consortium. Initial sequencing and analysis of the human genome. Nature.

[B6] Pruitt KDTT, Maglott DR (2005). NCBI Reference Sequence (RefSeq): a curated non-redundant sequence database of genomes, transcripts, and proteins. Nucleic Acids Research.

[B7] Taira M, Iizasa T, Shimada H, Kudoh J, Shimizu N, Tatibana M (1990). A human testis-specific mRNA for phosphoribosylpyrophosphate synthetase that initiates from a non-AUG codon. Journal of Biological Chemistry.

[B8] Bernards A, de la Monte SM (1990). The ltk receptor tyrosine kinase is expressed in pre-B lymphocytes and cerebral neurons and uses a non-AUG translational initiator. The EMBO Journal.

[B9] Hann SR, Dixit M, Sears RC, Sealy L (1994). The alternatively initiated c-Myc proteins differentially regulate transcription through a noncanonical DNA-binding site. Genes & Development.

[B10] Grana X, Claudio PP, De Luca A, Sang N, Giordano A (1994). PISSLRE, a human novel CDC2-related protein kinase. Oncogene.

[B11] Palmiter RD, Cole TB, Findley SD (1996). ZnT-2, a mammalian protein that confers resistance to zinc by facilitating vesicular sequestration. The EMBO Journal.

[B12] Stewart AF, Richard CW, Suzow J, Stephan D, Weremowicz S, Morton CC, Adra CN (1996). Cloning of human RTEF-1, a transcriptional enhancer factor-1-related gene preferentially expressed in skeletal muscle: evidence for an ancient multigene family. Genomics.

[B13] Imataka H, Olsen HS, Sonenberg N (1997). A new translational regulator with homology to eukaryotic translation initiation factor 4G. The EMBO Journal.

[B14] Dodds DC, Omeis IA, Cushman SJ, Helms JA, Perin MS (1997). Neuronal pentraxin receptor, a novel putative integral membrane pentraxin that interacts with neuronal pentraxin 1 and 2 and taipoxin-associated calcium-binding protein 49. Journal of Biological Chemistry.

[B15] Takayama S, Krajewski S, Krajewska M, Kitada S, Zapata JM, Kochel K, Knee D, Scudiero D, Tudor G, Miller GJ, Miyashita T, Yamada M, Reed JC (1998). Expression and location of Hsp70/Hsc-binding anti-apoptotic protein BAG-1 and its variants in normal tissues and tumor cell lines. Cancer Res.

[B16] Yang X, Chernenko G, Hao Y, Ding Z, Pater MM, Pater A, Tang SC (1998). Human BAG-1/RAP46 protein is generated as four isoforms by alternative translation initiation and overexpressed in cancer cells. Oncogene.

[B17] Bertilsson G, Heidrich J, Svensson K, Asman M, Jendeberg L, Sydow-Backman M, Ohlsson R, Postlind H, Blomquist P, Berkenstam A (1998). Identification of a human nuclear receptor defines a new signaling pathway for CYP3A induction. Proc Natl Acad Sci U S A.

[B18] Arnaud E, Touriol C, Boutonnet C, Gensac MC, Vagner S, Prats H, Prats AC (1999). A new 34-kilodalton isoform of human fibroblast growth factor 2 is cap dependently synthesized by using a non-AUG start codon and behaves as a survival factor. Molecular and Cellular Biology.

[B19] Dolstra H, Fredrix H, Maas F, Coulie PG, Brasseur F, Mensink E, Adema GJ, de Witte TM, Figdor CG, Wiel-van Kemenade E van de (1999). A human minor histocompatibility antigen specific for B cell acute lymphoblastic leukemia. Journal of Experimental Medicine.

[B20] Shaughnessy JD, Largaespada DA, Tian E, Fletcher CF, Cho BC, Vyas P, Jenkins NA, Copeland NG (1999). Mrvi1, a common MRV integration site in BXH2 myeloid leukemias, encodes a protein with homology to a lymphoid-restricted membrane protein Jaw1. Oncogene.

[B21] Jiang SW, Wu K, Eberhardt NL (1999). Human placental TEF-5 transactivates the human chorionic somatomammotropin gene enhancer. Molecular endocrinology.

[B22] Thebault S, Gachon F, Lemasson I, Devaux C, Mesnard JM (2000). Molecular cloning of a novel human I-mfa domain-containing protein that differently regulates human T-cell leukemia virus type I and HIV-1 expression. Journal of Biological Chemistry.

[B23] Burgess DL, Gefrides LA, Foreman PJ, Noebels JL (2001). A cluster of three novel Ca2+ channel gamma subunit genes on chromosome 19q13.4: evolution and expression profile of the gamma subunit gene family. Genomics.

[B24] Flomerfelt FA, Kim MG, Schwartz RH (2000). Spatial, a gene expressed in thymic stromal cells, depends on three-dimensional thymus organization for its expression. Genes Immun.

[B25] Williams RT, Manji SS, Parker NJ, Hancock MS, Van Stekelenburg L, Eid JP, Senior PV, Kazenwadel JS, Shandala T, Saint R, Smith PJ, Dziadek MA (2001). Identification and characterization of the STIM (stromal interaction molecule) gene family: coding for a novel class of transmembrane proteins. The Biochemical Journal.

[B26] Peretti M, Villard J, Barras E, Zufferey M, Reith W (2001). Expression of the three human major histocompatibility complex class II isotypes exhibits a differential dependence on the transcription factor RFXAP. Mol Cell Biol.

[B27] Uhlmann-Schiffler H, Rossler OG, Stahl H (2002). The mRNA of DEAD box protein p72 is alternatively translated into an 82-kDa RNA helicase. The Journal of Biological Chemistry.

[B28] Short JD, Pfarr CM (2002). Translational regulation of the JunD messenger RNA. J Biol Chem.

[B29] Sapetschnig A, Koch F, Rischitor G, Mennenga T, Suske G (2004). Complexity of translationally controlled transcription factor Sp3 isoform expression. J Biol Chem.

[B30] Shiokawa D, Shika Y, Saito K, Yamazaki K, Tanuma S (2005). Physical and biochemical properties of mammalian DNase X proteins: non-AUG translation initiation of porcine and bovine mRNAs for DNase X. The Biochemical Journal.

[B31] Kozak M (1991). An Analysis of Vertebrate mRNA Sequences: Intimations of Translational Control. Journal of Cell Biology.

[B32] Crooks GE, Hon G, Chandonia JM, Brenner SE (2004). Web Logo: A sequence logo generator. Genome Research.

[B33] Carmela MignoneFG, Sabino Liuni, Graziano Pesole (2002). Untranslated Regions on mRNAs. Genome Biology.

[B34] Stefanie KosMD, George Reid, Frank Gannon (2002). Upstream Open Reading Frames Regulate the Translation of the Multiple mRNA Variants of the Estrogen Receptor a. Journal of Biological Chemistry.

[B35] Kozak M (2002). Pushing the limits of the scanning mechanism for initiation of translation. Gene.

[B36] Boyd L, Thummel C (1993). Selection of CUG and AUG initiator codons for Drosophila E74A translation depends on downstream sequences. Proceedings of the National Academy of Sciences of the United States of America.

[B37] O'Donnell S, Janssen G (2001). The Initiation Codon Affects Ribosome Binding and Translational Efficiency in *Escherichia coli *of *c*I mRNA with or without the 5' Untranslated Leader. J Bacteriol.

[B38] Morris D, Geballe A (2000). Upstream Open Reading Frames as Regulators of mRNA Translation. Molecular and Cellular Biology.

[B39] Jackson RJ (2005). Alternative Mechanisms of Initiating Translation of Mammalian mRNAs. Biochem Soc Trans.

[B40] Kozak M (2001). Constraints on reinitiation of translation in mammals. Nucleic Acids Research.

[B41] Hatzigeorgiou A (2002). Translation initiation start prediction in human cDNAs with high accuracy. Bioinformatics.

[B42] Zeng F, Yap R (2002). Using Feature Generation and Feature Selection for Accurate Prediction of Translation Initiation Sites. Genome Informatics.

[B43] Benson DA, Karsch-Mizrachi I, Lipman DJ, Ostell J, Wheller DL (2006). GenBank. Nucleic Acids Research.

[B44] Carmela MignoneFG, Sabino Liuni, Graziano Pesole (2005). UTRdb and UTRsite: a collection of sequences and regulatory emotifs of the untranslated regions of eukaryotic mRNAs. Nucleic Acids Research.

[B45] Zhang J, Long M, Li L (2005). Translational effects of differential codon usage among intragenic domains of new genes in *Drosophilia*. Biochimica et Biophysica Acta.

[B46] Qin H, Wu WB, Comeron J, Kreitman M, Li W (2004). Intragenic Spatial Patterns of Codon Usage Bias in Prokaryotic and Eukaryotic Genomes. Genetics.

[B47] Gustafsson C, Govindarajan S, Minshull J (2004). Codon Bias and heterologous protein expression. Trends in Biotechnology.

[B48] Peden J (1997). CodonW: A Codon Usage Analysis Program. http://www.bio.net/bionet/mm/bionews/1997-July/003992.html.

[B49] Fuglsang A (2006). Estimating the "Effective Number of Codons": The Wright Way of Determining Codon Homozygosity Leads to Superior Estimates. Genetics.

[B50] Zuker AM, Mathews B, Turner C Algorithms and Thermodynamics for RNA Secondary Structure Prediction: A Practical Guide. http://www.bioinfo.rpi.edu/~zukerm/seqanal/.

[B51] Dimitrov RA, Zuker M (2005). Prediction of hybridization and melting for double-stranded nucleic acids. Biophysical Journal.

[B52] Witten I, Frank E (2005). Data Mining: Practical machine learning tools and techniques.

[B53] Bajic V, Tang S, Han H, Brusic V, Hatzigeorgiou A (2002). Artificial Neural Networks Based Systems for Recognition of Genomic Signals and Regions: A Review. J Informatica.

