# Supplementary material for: Bioinformatic analyses of mammalian 5'-UTR sequence properties of mRNAs predicts alternative translation initiation sites
Source: BMC Bioinformatics. 2008 May 8;9:232. doi: 10.1186/1471-2105-9-232 (PMC2396638; doi:10.1186/1471-2105-9-232)
Supplement: Additional file 1 — Negative set of Mammalian AUG, non-ATIS, mRNA Sequences. A set of 500 sequences were selected from Mammalian species that translate with one or more AUG start sites. These sequences were selected from the validated RefSeq database. [file 1471-2105-9-232-S1.pdf]

Supplemental Table 1: Negative set of Mammalian AUG, non-aTIS, mRNA Sequences

Accession	Protein	Species
NM_000055.2	butyrylcholinesterase (BCHE).	Homo sapiens
NM_000101.2	cytochrome b-245, alpha polypeptide (CYBA).	Homo sapiens
NM_000145.2	follicle stimulating hormone receptor (FSHR), tv 1.	Homo sapiens
NM_000202.3	iduronate 2-sulfatase (Hunter syndrome) (IDS), tv 1.	Homo sapiens
NM_000529.2	melanocortin 2 receptor (adrenocorticotropic hormone) (MC2R).	Homo sapiens
NM_000585.2	interleukin 15 (IL15), tv 3.	Homo sapiens
NM_000734.2	CD247 molecule (CD247), tv 2.	Homo sapiens
NM_000749.3	cholinergic receptor, nicotinic, beta 3 (CHRNB3).	Homo sapiens
NM_000812.2	gamma-aminobutyric acid (GABA) A receptor, beta 1 (GABRB1).	Homo sapiens
NM_000983.3	ribosomal protein L22 (RPL22).	Homo sapiens
NM_001001343.2	MGC27121 gene (MGC27121).	Homo sapiens
NM_001001798.2	Atpase, class VI, type 11C (Atp11c), tv 2.	Mus musculus
NM_001001986.1	RIKEN cDNA 8430427H17 gene (8430427H17Rik).	Mus musculus
NM_001002023.2	sarcoglycan, epsilon (Sgce).	Rattus norvegicus
NM_001005214.2	leucine rich repeat containing 52 (LRRC52).	Homo sapiens
NM_001005847.1	aspartylglucosaminidase (Aga).	Mus musculus
NM_001006622.1	WD repeat domain 33 (WDR33), tv 2.	Homo sapiens
NM_001006997.1	sphingomyelin phosphodiesterase 1, acid lysosomal (Smpd1).	Rattus norvegicus
NM_001007559.1	synovial sarcoma translocation, chromosome 18 (SS18), tv 1.	Homo sapiens
NM_001007561.1	immunity-related GTPase family, Q (IRGQ).	Homo sapiens
NM_001008389.1	uromodulin (uromucoid, Tamm-Horsfall glycoprotein) (UMOD), tv 2.	Homo sapiens
NM_001008711.1	RNA binding protein with multiple splicing (RBPMS), tv 2.	Homo sapiens
NM_001008783.1	solute carrier family 35, member D3 (SLC35D3).	Homo sapiens
NM_001009197.2	interferon alpha 7 (IFNA7).	Felis catus
NM_001009210.1	parathyroid hormone (PTH).	Felis catus
NM_001009211.1	interleukin 6 (IL6).	Felis catus
NM_001009227.1	colony stimulating factor 3 (granulocyte) (CSF3).	Felis catus
NM_001009233.1	Fel d 4 allergen (LOC493739).	Felis catus
NM_001009244.1	natriuretic peptide precursor B (NPPB).	Felis catus
NM_001009249.1	carboxylesterase 1 (CES1).	Felis catus
NM_001009262.1	melanoma antigen 2 (LOC493792).	Felis catus
NM_001009269.1	erythropoietin (EPO).	Felis catus
NM_001009278.1	motilin (MLN).	Felis catus
NM_001009281.1	interleukin 8 (IL8).	Felis catus
NM_001009282.1	BCL2-associated X protein (BAX).	Felis catus
NM_001009294.1	tumor protein p53 (TP53).	Felis catus
NM_001009300.1	prostaglandin D2 synthase (PTGDS).	Felis catus
NM_001009302.1	feline leukemia virus subgroup C cellular receptor (FLVCR).	Felis catus
NM_001009303.1	growth associated protein 43 (GAP43).	Felis catus
NM_001009334.1	glutamate transporter GLT1 (GLT1).	Felis catus
NM_001009337.1	growth hormone (GH).	Felis catus
NM_001009338.1	caspase 3 (CASP3).	Felis catus
NM_001009343.1	KIT ligand (KITLG).	Felis catus
NM_001009364.1	butyrylcholinesterase (BCHE).	Felis catus
NM_001009368.1	melanoma antigen MAGE (LOC493964).	Felis catus
NM_001009371.1	cytochrome P450, family 17, subfamily A, polypeptide 1 (CYP17A1).	Felis catus
NM_001009374.1	myeloid cell leukemia sequence 1 (MCL1).	Felis catus
NM_001009386.1	endothelin 1 (EDN1).	Felis catus
NM_001009826.1	chemokine (C-X-C motif) receptor 4 (CXCR4).	Felis catus
NM_001009827.1	chemokine (C-C motif) ligand 5 (CCL5).	Felis catus
NM_001009836.1	thiopurine S-methyltransferase (TPMT).	Felis catus
NM_001009845.2	interleukin 5 (IL5).	Felis catus
NM_001009847.1	chemokine (C-X-C motif) ligand 12 (CXCL12).	Felis catus
NM_001009850.1	leptin (LEP).	Felis catus
NM_001009852.1	bladder cancer associated protein (BLCAP).	Felis catus
NM_001009853.1	ghrelin (GHRL).	Felis catus
NM_001009868.1	pregnancy-associated glycoprotein (LOC493954).	Felis catus
NM_001009931.1	hornerin (HRNR).	Homo sapiens
NM_001010891.2	metaxin 3 (MTX3).	Homo sapiens

Supplemental Table 1: Negative set of Mammalian AUG, non-aTIS, mRNA Sequences

Accession	Protein	Species
NM_001010908.1	complement component 1, q subcomponent-like 3 (C1QL3).	Homo sapiens
NM_001012023.1	erythrocyte protein band 4.1-like 5 (Epb4.1l5).	Rattus norvegicus
NM_001012740.1	cerebellin 2 precursor protein (Cbln2).	Rattus norvegicus
NM_001013143.1	telomeric repeat binding factor 2, interacting protein (Terf2ip).	Rattus norvegicus
NM_001013413.1	amyotrophic lateral sclerosis 2 (juvenile) homolog (human) (Als2).	Rattus norvegicus
NM_001013825.2	predicted gene, EG435366 (EG435366).	Mus musculus
NM_001013843.1	SAFB-like, transcription modulator (SLTM), tv 2.	Homo sapiens
NM_001013908.1	RIKEN cDNA 2410002O22 gene (RGD1306583).	Rattus norvegicus
NM_001015036.1	sialyltransferase 7F (Siat7F).	Rattus norvegicus
NM_001020819.1	myeloid-associated differentiation marker (MYADM), tv 3.	Homo sapiens
NM_001024163.1	caveolin 1 (CAV1).	Felis catus
NM_001024279.1	lymphocyte antigen 96 (Ly96).	Rattus norvegicus
NM_001024607.1	hypothetical LOC154907 (LOC154907).	Homo sapiens
NM_001024732.1	mitochondrial trans-2-enoyl-CoA reductase (MECR), tv 2.	Homo sapiens
NM_001024940.1	tripartite motif-containing 17 (TRIM17), tv 2.	Homo sapiens
NM_001025026.1	signal-transducing adaptor protein-2 (Stap2).	Rattus norvegicus
NM_001025723.1	mitochondrial translational release factor 1-like (LOC361473).	Rattus norvegicus
NM_001029853.1	phosphodiesterase 8B (PDE8B), tv 4.	Homo sapiens
NM_001030049.1	kallikrein-related peptidase 3 (KLK3), tv 5.	Homo sapiens
NM_001031721.2	zinc finger protein 613 (ZNF613), tv 1.	Homo sapiens
NM_001032287.1	nuclear receptor subfamily 2, group C, member 1 (NR2C1), tv 2.	Homo sapiens
NM_001033582.1	protein kinase C, zeta (PRKCZ), tv 3.	Homo sapiens
NM_001034740.1	LSM8 homolog, U6 small nuclear RNA associated (S. cerevisiae) (LSM8).	Bos taurus
NM_001034795.1	galactose mutarotase (aldose 1-epimerase) (GALM).	Bos taurus
NM_001037139.1	protocadherin gamma a2 (Pcdhga2).	Rattus norvegicus
NM_001037174.1	ADP-ribosylation factor-like 5A (ARL5A), tv 3.	Homo sapiens
NM_001038213.2	tRNA (5-methylaminomethyl-2-thiouridylate)-methyltransferase 1 (MGC133954).	Bos taurus
NM_001039130.1	arachidonate 15-lipoxygenase, type B (ALOX15B), tv a.	Homo sapiens
NM_001039140.1	chromosome 20 open reading frame 27 (C20orf27).	Homo sapiens
NM_001039141.1	TRIO and F-actin binding protein (TRIOBP), tv 6.	Homo sapiens
NM_001039467.1	regulator of G-protein signalling 19 (RGS19), tv 2.	Homo sapiens
NM_001039936.1	chimerin (chimaerin) 2 (CHN2), tv 1.	Homo sapiens
NM_001039941.2	cysteine-rich perinuclear theca 8 (Cypt8).	Mus musculus
NM_001039942.2	cysteine-rich perinuclear theca 9 (Cypt9).	Mus musculus
NM_001039943.2	cysteine-rich perinuclear theca 7 (Cypt7).	Mus musculus
NM_001039944.2	cysteine-rich perinuclear theca 10 (Cypt10).	Mus musculus
NM_001040139.1	chemokine-like factor (CKLF), tv 6.	Homo sapiens
NM_001042402.1	N-acylsphingosine amidohydrolase (acid ceramidase)-like (ASAHL), tv 2.	Homo sapiens
NM_001042498.2	solute carrier family 35 (UDP-galactose transporter), member A2 (SLC35A2), tv 3.	Homo sapiens
NM_001042563.1	alkaline phosphatase (ALPL).	Felis catus
NM_001042566.1	alanine-glyoxylate aminotransferase (AGXT).	Felis catus
NM_001042584.1	CD1e molecule (CD1E), tv 3.	Homo sapiens
NM_001042595.1	transmembrane protein 91 (TMEM91).	Homo sapiens
NM_001042724.1	poliovirus receptor-related 2 (herpesvirus entry mediator B) (PVRL2), tv delta.	Homo sapiens
NM_001043337.1	interleukin 2 (IL2).	Felis catus
NM_001043341.1	prolactin (PRL).	Felis catus
NM_001046420.1	Protein FAM26B (MGC137966).	Bos taurus
NM_001046467.1	hypothetical LOC540061 (LOC540061).	Bos taurus
NM_001046512.1	tetraspanin 12 (TSPAN12).	Bos taurus
NM_001048153.1	major allergen I, polypeptide chain 1 (LOC677877).	Felis catus
NM_001048154.1	major allergen I, polypeptide chain 2 (LOC677879).	Felis catus
NM_001048157.1	ribosomal protein L41 (RPL41).	Felis catus
NM_001048183.1	phosphatase and actin regulator 4 (PHACTR4), tv 1.	Homo sapiens
NM_001048195.1	regulator of chromosome condensation 1 (RCC1), tv 2.	Homo sapiens
NM_001048265.1	chromosome 9 open reading frame 116 (C9orf116), tv 1.	Homo sapiens
NM_001076067.1	26S proteasome non-ATPase regulatory subunit 14(MAD1) (MGC139782).	Bos taurus
NM_001076418.2	hypothetical protein LOC616333 (FAM79A).	Bos taurus
NM_001076520.1	TBC1 domain family, member 22A (LOC618784).	Bos taurus
NM_001076882.1	Zinc finger protein 215 (BWSCR2 associated zinc-finger protein 2) (BAZ 2) (MGC142562).	Bos taurus

Supplemental Table 1: Negative set of Mammalian AUG, non-aTIS, mRNA Sequences

Accession	Protein	Species
NM_001077245.1	oocyte specific protein COEP19 (COEP19).	Felis catus
NM_001077366.1	protein-O-mannosyltransferase 1 (POMT1), tv 3.	Homo sapiens
NM_001077478.1	nuclear receptor subfamily 1, group I, member 3 (NR1I3), tv 7.	Homo sapiens
NM_001077673.1	RAD18 homolog (S. cerevisiae) (predicted) (Rad18_predicted).	Rattus norvegicus
NM_001077685.1	centaurin, gamma-like family, member 4 (CTGLF4).	Homo sapiens
NM_001079860.1	G protein-coupled receptor 64 (GPR64), tv 3.	Homo sapiens
NM_001080141.1	cancer/testis CT47 family, member 6 (CT47.6).	Homo sapiens
NM_001080522.1	KIAA1345 protein (KIAA1345).	Homo sapiens
NM_001080975.1	RALBP1 associated Eps domain containing 2 (REPS2), tv 2.	Homo sapiens
NM_001081350.1	nucleolar protein 8 (Nol8).	Mus musculus
NM_001081550.1	THO complex 2 (THOC2), tv 1.	Homo sapiens
NM_001082969.1	chromosome 11 open reading frame 57 (C11orf57), tv 2.	Homo sapiens
NM_001083474.1	eukaryotic translation initiation factor EIF2B subunit 3 (MGC142785).	Bos taurus
NM_001083537.1	family with sequence similarity 86, member B1 (FAM86B1), tv 1.	Homo sapiens
NM_001097638.1	fucosyltransferase 2 (secretor status included) (FUT2), tv 2.	Homo sapiens
NM_001098130.1	amphiphysin I (MGC140160).	Bos taurus
NM_001098200.1	G protein-coupled receptor 18 (GPR18), tv 2.	Homo sapiens
NM_001141.2	arachidonate 15-lipoxygenase, type B (ALOX15B), tv d.	Homo sapiens
NM_001150.1	alanyl (membrane) aminopeptidase (ANPEP).	Homo sapiens
NM_001299.4	calponin 1, basic, smooth muscle (CNN1).	Homo sapiens
NM_001304.3	carboxypeptidase D (CPD).	Homo sapiens
NM_001384.4	DPH2 homolog (S. cerevisiae) (DPH2), tv 1.	Homo sapiens
NM_001400.3	endothelial differentiation, sphingolipid G-protein-coupled receptor, 1 (EDG1).	Homo sapiens
NM_001464.3	ADAM metallopeptidase domain 2 (fertilin beta) (ADAM2).	Homo sapiens
NM_001502.2	glycoprotein 2 (zymogen granule membrane) (GP2), tv 2.	Homo sapiens
NM_001537.2	heat shock factor binding protein 1 (HSBP1).	Homo sapiens
NM_001542.2	immunoglobulin superfamily, member 3 (IGSF3), tv 1.	Homo sapiens
NM_001559.2	interleukin 12 receptor, beta 2 (IL12RB2).	Homo sapiens
NM_001807.3	carboxyl ester lipase (bile salt-stimulated lipase) (CEL).	Homo sapiens
NM_001821.3	choroideremia-like (Rab escort protein 2) (CHML).	Homo sapiens
NM_001888.2	crystallin, mu (CRYM), tv 1.	Homo sapiens
NM_001911.2	cathepsin G (CTSG).	Homo sapiens
NM_001919.2	dodecenoyl-Coenzyme A delta isomerase (DCI), nuclear gene encoding mitochondrial protein.	Homo sapiens
NM_001957.1	endothelin receptor type A (EDNRA).	Homo sapiens
NM_001980.2	syntaxin 2 (STX2), tv 1.	Homo sapiens
NM_002001.2	Fc fragment of IgE, high affinity I, receptor for; alpha polypeptide (FCER1A).	Homo sapiens
NM_002021.1	flavin containing monooxygenase 1 (FMO1).	Homo sapiens
NM_002204.1	integrin, alpha 3 (antigen CD49C, alpha 3 subunit of VLA-3 receptor) (ITGA3), tv a.	Homo sapiens
NM_002285.2	AF4/FMR2 family, member 3 (AFF3), tv 1.	Homo sapiens
NM_002356.4	myristoylated alanine-rich protein kinase C substrate (MARCKS).	Homo sapiens
NM_002441.3	mutS homolog 5 (E. coli) (MSH5), tv 3.	Homo sapiens
NM_002715.2	protein phosphatase 2 (formerly 2A), catalytic subunit, alpha isoform (PPP2CA).	Homo sapiens
NM_002740.5	protein kinase C, iota (PRKCI).	Homo sapiens
NM_002750.2	mitogen-activated protein kinase 8 (MAPK8), tv JNK1-a1.	Homo sapiens
NM_002788.2	proteasome (prosome, macropain) subunit, alpha type, 3 (PSMA3), tv 1.	Homo sapiens
NM_002839.2	protein tyrosine phosphatase, receptor type, D (PTPRD), tv 1.	Homo sapiens
NM_002872.3	ras-related C3 botulinum toxin substrate 2 (rho family, small GTP binding protein Rac2) (RAC2).	Homo sapiens
NM_002993.2	chemokine (C-X-C motif) ligand 6 (granulocyte chemotactic protein 2) (CXCL6).	Homo sapiens
NM_003015.2	secreted frizzled-related protein 5 (SFRP5).	Homo sapiens
NM_003020.2	secretogranin V (7B2 protein) (SCG5).	Homo sapiens
NM_003022.1	SH3 domain binding glutamic acid-rich protein like (SH3BGRL).	Homo sapiens
NM_003125.2	small proline-rich protein 1B (cornifin) (SPRR1B).	Homo sapiens
NM_003430.2	zinc finger protein 91 (ZNF91).	Homo sapiens
NM_003477.1	pyruvate dehydrogenase complex, component X (PDHX).	Homo sapiens
NM_003488.3	A kinase (PRKA) anchor protein 1 (AKAP1), nuclear gene encoding mitochondrial protein.	Homo sapiens
NM_003577.2	undifferentiated embryonic cell transcription factor 1 (UTF1).	Homo sapiens
NM_003589.2	cullin 4A (CUL4A), tv 2.	Homo sapiens
NM_003644.2	growth arrest-specific 7 (GAS7), tv a.	Homo sapiens
NM_003671.3	CDC14 cell division cycle 14 homolog B (S. cerevisiae) (CDC14B), tv 1.	Homo sapiens

Supplemental Table 1: Negative set of Mammalian AUG, non-aTIS, mRNA Sequences

Accession	Protein	Species
NM_003743.4	nuclear receptor coactivator 1 (NCOA1), tv 1.	Homo sapiens
NM_003806.1	harakiri, BCL2 interacting protein (contains only BH3 domain) (HRK).	Homo sapiens
NM_003882.2	WNT1 inducible signaling pathway protein 1 (WISP1), tv 1.	Homo sapiens
NM_004014.1	dystrophin (muscular dystrophy, Duchenne and Becker types) (DMD), tv Dp116.	Homo sapiens
NM_004280.3	eukaryotic translation elongation factor 1 epsilon 1 (EEF1E1).	Homo sapiens
NM_004313.3	arrestin, beta 2 (ARRB2), tv 1.	Homo sapiens
NM_004352.1	cerebellin 1 precursor (CBLN1).	Homo sapiens
NM_004487.3	golgi autoantigen, golgin subfamily b, macrogolgin (with transmembrane signal), 1 (GOLGB1).	Homo sapiens
NM_004727.2	solute carrier family 24 (sodium/potassium/calcium exchanger), member 1 (SLC24A1).	Homo sapiens
NM_004776.2	UDP-Gal:betaGlcNAc beta 1,4- galactosyltransferase, polypeptide 5 (B4GALT5).	Homo sapiens
NM_004804.2	cytosolic iron-sulfur protein assembly 1 homolog (S. cerevisiae) (CIAO1).	Homo sapiens
NM_005201.2	chemokine (C-C motif) receptor 8 (CCR8).	Homo sapiens
NM_005221.5	distal-less homeobox 5 (DLX5).	Homo sapiens
NM_005234.3	nuclear receptor subfamily 2, group F, member 6 (NR2F6).	Homo sapiens
NM_005265.2	gamma-glutamyltransferase 1 (GGT1), tv 1.	Homo sapiens
NM_005343.2	v-Ha-ras Harvey rat sarcoma viral oncogene homolog (HRAS), tv 1.	Homo sapiens
NM_005385.3	natural killer-tumor recognition sequence (NKTR).	Homo sapiens
NM_005493.2	RAN binding protein 9 (RANBP9).	Homo sapiens
NM_005507.2	cofilin 1 (non-muscle) (CFL1).	Homo sapiens
NM_005568.2	LIM homeobox 1 (LHX1).	Homo sapiens
NM_005625.3	syndecan binding protein (syntenin) (SDCBP), tv 1.	Homo sapiens
NM_005663.2	Wolf-Hirschhorn syndrome candidate 2 (WHSC2).	Homo sapiens
NM_005762.2	tripartite motif-containing 28 (TRIM28).	Homo sapiens
NM_005842.2	sprouty homolog 2 (Drosophila) (SPRY2).	Homo sapiens
NM_005888.2	solute carrier family 25 member 3 (SLC25A3), nuclear gene encoding mitochondrial protein, tv 1.	Homo sapiens
NM_005981.3	tetraspanin 31 (TSPAN31).	Homo sapiens
NM_006002.3	ubiquitin carboxyl-terminal esterase L3 (ubiquitin thiolesterase) (UCHL3).	Homo sapiens
NM_006194.1	paired box gene 9 (PAX9).	Homo sapiens
NM_006203.3	phosphodiesterase 4D, cAMP-specific (phosphodiesterase E3 dunce homolog, Drosophila) (PDE4D)	Homo sapiens
NM_006258.2	protein kinase, cGMP-dependent, type I (PRKG1).	Homo sapiens
NM_006279.2	ST3 beta-galactoside alpha-2,3-sialyltransferase 3 (ST3GAL3), tv 10.	Homo sapiens
NM_006289.2	talin 1 (TLN1).	Homo sapiens
NM_006533.2	melanoma inhibitory activity (MIA).	Homo sapiens
NM_006551.3	secretoglobin, family 1D, member 2 (SCGB1D2).	Homo sapiens
NM_006600.2	nuclear distribution gene C homolog (A. nidulans) (NUDC).	Homo sapiens
NM_006693.2	cleavage and polyadenylation specific factor 4, 30kDa (CPSF4), tv 1.	Homo sapiens
NM_006753.3	surfeit 6 (SURF6).	Homo sapiens
NM_006830.2	ubiquinol-cytochrome c reductase, 6.4kDa subunit (UQCR).	Homo sapiens
NM_006908.3	ras-related C3 botulinum toxin substrate 1 (RAC1), tv Rac1.	Homo sapiens
NM_007074.2	coronin, actin binding protein, 1A (CORO1A).	Homo sapiens
NM_007174.1	citron (rho-interacting, serine/threonine kinase 21) (CIT).	Homo sapiens
NM_007208.2	mitochondrial ribosomal protein L3 (MRPL3), nuclear gene encoding mitochondrial protein.	Homo sapiens
NM_007297.2	breast cancer 1, early onset (BRCA1), tv BRCA1-delta2-10.	Homo sapiens
NM_007324.2	zinc finger, FYVE domain containing 9 (ZFYVE9), tv 1.	Homo sapiens
NM_007347.3	adaptor-related protein complex 4, epsilon 1 subunit (AP4E1).	Homo sapiens
NM_007870.3	deoxyribonuclease 1-like 3 (Dnase1l3).	Mus musculus
NM_009495.1	vomeronasal 2, receptor, 9 (V2r9).	Mus musculus
NM_009590.2	amine oxidase, copper containing 2 (retina-specific) (AOC2), tv 2.	Homo sapiens
NM_010009.1	cytochrome P450, family 27, subfamily b, polypeptide 1 (Cyp27b1).	Mus musculus
NM_010145.2	epoxide hydrolase 1, microsomal (Ephx1).	Mus musculus
NM_010491.1	islet amyloid polypeptide (Iapp).	Mus musculus
NM_010770.2	matrilin 3 (Matn3).	Mus musculus
NM_011055.2	phosphodiesterase 3B, cGMP-inhibited (Pde3b).	Mus musculus
NM_012146.1	double homeobox, 1 (DUX1).	Homo sapiens
NM_012148.2	double homeobox, 3 (DUX3).	Homo sapiens
NM_012149.2	double homeobox, 5 (DUX5).	Homo sapiens
NM_012193.2	frizzled homolog 4 (Drosophila) (FZD4).	Homo sapiens
NM_012326.2	microtubule-associated protein, RP/EB family, member 3 (MAPRE3).	Homo sapiens
NM_012544.1	angiotensin I converting enzyme (peptidyl-dipeptidase A) 1 (Ace).	Rattus norvegicus

Supplemental Table 1: Negative set of Mammalian AUG, non-aTIS, mRNA Sequences

Accession	Protein	Species
NM_013259.2	transgelin 3 (TAGLN3), tv 1.	Homo sapiens
NM_013290.4	PSMC3 interacting protein (PSMC3IP), tv 1.	Homo sapiens
NM_013299.3	SAC3 domain containing 1 (SAC3D1).	Homo sapiens
NM_013316.2	CCR4-NOT transcription complex, subunit 4 (CNOT4), tv 1.	Homo sapiens
NM_013412.1	RAB, member of RAS oncogene family-like 2A (RABL2A), tv 1.	Homo sapiens
NM_014078.4	mitochondrial ribosomal protein L13 (MRPL13), nuclear gene encoding mitochondrial protein.	Homo sapiens
NM_014096.2	solute carrier family 43, member 3 (SLC43A3).	Homo sapiens
NM_014155.3	zinc finger and BTB domain containing 44 (ZBTB44).	Homo sapiens
NM_014180.2	mitochondrial ribosomal protein L22 (MRPL22), nuclear gene encoding mitochondrial protein, tv 1	Homo sapiens
NM_014231.3	vesicle-associated membrane protein 1 (synaptobrevin 1) (VAMP1), tv 1.	Homo sapiens
NM_014254.1	transmembrane protein 5 (TMEM5).	Homo sapiens
NM_014410.4	clusterin-like 1 (retinal) (CLUL1), tv 1.	Homo sapiens
NM_014491.1	forkhead box P2 (FOXP2), tv 1.	Homo sapiens
NM_014615.1	KIAA0182 (KIAA0182).	Homo sapiens
NM_014622.4	loss of heterozygosity, 11, chromosomal region 2, gene A (LOH11CR2A), tv 1.	Homo sapiens
NM_014726.1	TBK1 binding protein 1 (TBKBP1).	Homo sapiens
NM_014922.4	NLR family, pyrin domain containing 1 (NLRP1), tv 2.	Homo sapiens
NM_015012.1	transmembrane protein 41B (TMEM41B).	Homo sapiens
NM_015296.1	dedicator of cytokinesis 9 (DOCK9).	Homo sapiens
NM_015310.3	pleckstrin and Sec7 domain containing 3 (PSD3), tv 1.	Homo sapiens
NM_015490.3	SEC31 homolog B (S. cerevisiae) (SEC31B).	Homo sapiens
NM_015527.2	TBC1 domain family, member 10B (TBC1D10B).	Homo sapiens
NM_015556.1	signal-induced proliferation-associated 1 like 1 (SIPA1L1).	Homo sapiens
NM_015621.2	coiled-coil domain containing 69 (CCDC69).	Homo sapiens
NM_015855.3	Wilms tumor upstream neighbor 1 (WIT1).	Homo sapiens
NM_015957.1	APAF1 interacting protein (APIP).	Homo sapiens
NM_016048.2	isochorismatase domain containing 1 (ISOC1).	Homo sapiens
NM_016098.1	brain protein 44-like (BRP44L).	Homo sapiens
NM_016179.1	transient receptor potential cation channel, subfamily C, member 4 (TRPC4).	Homo sapiens
NM_016343.3	centromere protein F, 350/400ka (mitosin) (CENPF).	Homo sapiens
NM_016545.4	immediate early response 5 (IER5).	Homo sapiens
NM_016651.5	dapper, antagonist of beta-catenin, homolog 1 (Xenopus laevis) (DACT1), tv 1.	Homo sapiens
NM_016653.2	sterile alpha motif and leucine zipper containing kinase AZK (ZAK), tv 1.	Homo sapiens
NM_016702.2	alanine-glyoxylate aminotransferase (Agxt).	Mus musculus
NM_016828.1	8-oxoguanine DNA glycosylase (OGG1), nuclear gene encoding mitochondrial protein, tv 2d.	Homo sapiens
NM_017029.1	neurofilament 3, medium (Nef3).	Rattus norvegicus
NM_017121.1	cathepsin J (Ctsj).	Rattus norvegicus
NM_017188.1	UNC-119 homolog (C. elegans) (Unc119).	Rattus norvegicus
NM_017519.1	AT rich interactive domain 1B (SWI1-like) (ARID1B), tv 1.	Homo sapiens
NM_017781.2	cytochrome P450, family 2, subfamily W, polypeptide 1 (CYP2W1).	Homo sapiens
NM_017815.1	chromosome 14 open reading frame 94 (C14orf94).	Homo sapiens
NM_017866.4	transmembrane protein 70 (TMEM70), tv 1.	Homo sapiens
NM_017880.1	chromosome 2 open reading frame 42 (C2orf42).	Homo sapiens
NM_018012.3	kinesin family member 26B (KIF26B).	Homo sapiens
NM_018022.1	transmembrane protein 51 (TMEM51).	Homo sapiens
NM_018034.2	WD repeat domain 70 (WDR70).	Homo sapiens
NM_018063.3	helicase, lymphoid-specific (HELLS).	Homo sapiens
NM_018076.2	armadillo repeat containing 4 (ARMC4).	Homo sapiens
NM_018109.2	PAP associated domain containing 1 (PAPD1).	Homo sapiens
NM_018219.1	coiled-coil domain containing 87 (CCDC87).	Homo sapiens
NM_018287.5	Rho GTPase activating protein 12 (ARHGAP12).	Homo sapiens
NM_018292.3	glutaminyl-tRNA synthase (glutamine-hydrolyzing)-like 1 (QRSL1).	Homo sapiens
NM_018426.1	transmembrane protein 63B (TMEM63B).	Homo sapiens
NM_018477.2	actin-related protein 10 homolog (S. cerevisiae) (ACTR10).	Homo sapiens
NM_018653.3	G protein-coupled receptor, family C, group 5, member C (GPRC5C), tv 2.	Homo sapiens
NM_018703.3	retinoblastoma binding protein 6 (RBBP6), tv 2.	Homo sapiens
NM_018958.2	chromosome 15 open reading frame 2 (C15orf2).	Homo sapiens
NM_018976.3	solute carrier family 38, member 2 (SLC38A2).	Homo sapiens
NM_019067.4	guanine nucleotide binding protein-like 3 (nucleolar)-like (GNL3L).	Homo sapiens

Supplemental Table 1: Negative set of Mammalian AUG, non-aTIS, mRNA Sequences

Accession	Protein	Species
NM_019353.1	thyroid peroxidase (Tpo).	Rattus norvegicus
NM_019629.1	proline-rich protein 15 (Prp15).	Mus musculus
NM_019884.2	glycogen synthase kinase 3 alpha (GSK3A).	Homo sapiens
NM_019892.3	inositol polyphosphate-5-phosphatase, 72 kDa (INPP5E).	Homo sapiens
NM_020132.4	1-acylglycerol-3-phosphate O-acyltransferase 3 (AGPAT3), tv 1.	Homo sapiens
NM_020151.2	START domain containing 7 (STARD7), tv 1.	Homo sapiens
NM_020342.1	solute carrier family 39 (zinc transporter), member 10 (SLC39A10).	Homo sapiens
NM_020399.2	golgi associated PDZ and coiled-coil motif containing (GOPC), tv 1.	Homo sapiens
NM_020672.1	S100 calcium binding protein A14 (S100A14).	Homo sapiens
NM_020680.3	SCY1-like 1 (S. cerevisiae) (SCYL1), tv A.	Homo sapiens
NM_020699.1	GATA zinc finger domain containing 2B (GATAD2B).	Homo sapiens
NM_020807.1	zinc finger protein 319 (ZNF319).	Homo sapiens
NM_021138.3	TNF receptor-associated factor 2 (TRAF2).	Homo sapiens
NM_021146.2	angiopoietin-like 7 (ANGPTL7).	Homo sapiens
NM_021502.1	trafficking protein particle complex 2-like (Trappc2l).	Mus musculus
NM_021847.2	vesicle-associated membrane protein, associated protein B and C (Vapb).	Rattus norvegicus
NM_021940.3	stromal membrane-associated protein 1 (SMAP1), tv 2.	Homo sapiens
NM_022127.1	solute carrier family 28 (sodium-coupled nucleoside transporter), member 3 (SLC28A3).	Homo sapiens
NM_022253.1	Csk binding protein (Cbp).	Rattus norvegicus
NM_022394.1	scaffold attachment factor B (Safb).	Rattus norvegicus
NM_022551.2	ribosomal protein S18 (RPS18).	Homo sapiens
NM_022564.2	matrix metallopeptidase 16 (membrane-inserted) (MMP16), tv 2.	Homo sapiens
NM_022575.2	vacuolar protein sorting 16 homolog (S. cerevisiae) (VPS16), tv 1.	Homo sapiens
NM_022586.1	neuropeptide FF-amide peptide precursor (Npff).	Rattus norvegicus
NM_022601.2	pyridoxine 5'-phosphate oxidase (Pnpo).	Rattus norvegicus
NM_022763.2	fibronectin type III domain containing 3B (FNDC3B).	Homo sapiens
NM_022783.1	DEP domain containing 6 (DEPDC6).	Homo sapiens
NM_022915.2	mitochondrial ribosomal protein L44 (MRPL44), nuclear gene encoding mitochondrial protein.	Homo sapiens
NM_023010.2	UPF3 regulator of nonsense transcripts homolog B (yeast) (UPF3B), tv 2.	Homo sapiens
NM_024042.2	meteorin, glial cell differentiation regulator (METRN).	Homo sapiens
NM_024139.2	calcium binding protein p22 (Chp).	Rattus norvegicus
NM_024318.2	leukocyte immunoglobulin-like receptor, subfamily A (with TM domain), member 6 (LILRA6).	Homo sapiens
NM_024334.1	transmembrane protein 43 (TMEM43).	Homo sapiens
NM_024488.1	CDK5 regulatory subunit associated protein 3 (Cdk5rap3).	Rattus norvegicus
NM_024527.4	abhydrolase domain containing 8 (ABHD8).	Homo sapiens
NM_024593.2	EF-hand calcium binding domain 1 (EFCAB1).	Homo sapiens
NM_024597.2	MAP7 domain containing 3 (MAP7D3).	Homo sapiens
NM_024604.1	hypothetical protein FLJ21908 (FLJ21908).	Homo sapiens
NM_024712.3	engulfment and cell motility 3 (ELMO3).	Homo sapiens
NM_024746.3	KIAA1822-like (KIAA1822L).	Homo sapiens
NM_024864.3	mitochondrial rRNA methyltransferase 1 homolog (S. cerevisiae) (MRM1).	Homo sapiens
NM_024908.2	WD repeat domain 76 (WDR76).	Homo sapiens
NM_025126.2	ring finger protein 34 (RNF34), tv 2.	Homo sapiens
NM_025195.2	tribbles homolog 1 (Drosophila) (TRIB1).	Homo sapiens
NM_025216.2	wingless-type MMTV integration site family, member 10A (WNT10A).	Homo sapiens
NM_025224.2	zinc finger and BTB domain containing 46 (ZBTB46).	Homo sapiens
NM_025606.3	mitochondrial ribosomal protein L16 (Mrpl16).	Mus musculus
NM_025750.2	RIKEN cDNA 4933417A18 gene (4933417A18Rik).	Mus musculus
NM_025949.2	ribosomal protein S6 kinase polypeptide 6 (Rps6ka6).	Mus musculus
NM_025997.2	RIKEN cDNA 2610204K14 gene (2610204K14Rik).	Mus musculus
NM_027165.2	cyclin-dependent kinase 3 (Cdk3).	Mus musculus
NM_030627.1	cytoplasmic polyadenylation element binding protein 4 (CPEB4).	Homo sapiens
NM_030882.2	apolipoprotein L, 2 (APOL2), tv alpha.	Homo sapiens
NM_030920.2	acidic (leucine-rich) nuclear phosphoprotein 32 family, member E (ANP32E).	Homo sapiens
NM_030936.2	ring finger protein 32 (RNF32).	Homo sapiens
NM_031004.2	smooth muscle alpha-actin (Acta2).	Rattus norvegicus
NM_031210.3	chromosome 14 open reading frame 156 (C14orf156).	Homo sapiens
NM_031446.3	chromosome 18 open reading frame 21 (C18orf21).	Homo sapiens
NM_031585.1	phospholipase A2, group IB (Pla2g1b).	Rattus norvegicus

Supplemental Table 1: Negative set of Mammalian AUG, non-aTIS, mRNA Sequences

Accession	Protein	Species
NM_031689.1	crystallin, beta A4 (Cryba4).	Rattus norvegicus
NM_031690.1	crystallin, beta B3 (Crybb3).	Rattus norvegicus
NM_031729.1	protein phosphatase 5, catalytic subunit (Ppp5c).	Rattus norvegicus
NM_031781.1	amyloid beta (A4) precursor protein-binding, family A, member 3 (Apba3).	Rattus norvegicus
NM_031806.1	G protein-coupled receptor 6 (Gpr6).	Rattus norvegicus
NM_031864.1	protocadherin alpha 12 (PCDHA12), tv 2.	Homo sapiens
NM_031917.2	angiopoietin-like 6 (ANGPTL6).	Homo sapiens
NM_031956.2	tetratricopeptide repeat domain 29 (TTC29).	Homo sapiens
NM_031969.1	calmodulin 1 (Calm1).	Rattus norvegicus
NM_032014.2	mitochondrial ribosomal protein S24 (MRPS24), nuclear gene encoding mitochondrial protein.	Homo sapiens
NM_032351.3	mitochondrial ribosomal protein L45 (MRPL45), nuclear gene encoding mitochondrial protein.	Homo sapiens
NM_032433.2	zinc finger protein 333 (ZNF333).	Homo sapiens
NM_032458.2	PHD finger protein 6 (PHF6), tv 2.	Homo sapiens
NM_032479.2	mitochondrial ribosomal protein L36 (MRPL36), nuclear gene encoding mitochondrial protein.	Homo sapiens
NM_032553.1	G protein-coupled receptor 174 (GPR174).	Homo sapiens
NM_032615.2	membrane interacting protein of RGS16 (Mir16).	Rattus norvegicus
NM_032683.2	hypothetical protein MGC12972 (FKSG24).	Homo sapiens
NM_032837.1	family with sequence similarity 104, member A (FAM104A).	Homo sapiens
NM_032932.3	RAB11 family interacting protein 4 (class II) (RAB11FIP4).	Homo sapiens
NM_032993.2	nucleolar protein family A, member 1 (H/ACA small nucleolar RNPs) (NOLA1), tv 2.	Homo sapiens
NM_033036.2	galactose-3-O-sulfotransferase 3 (GAL3ST3).	Homo sapiens
NM_033056.3	protocadherin 15 (PCDH15).	Homo sapiens
NM_033135.3	platelet derived growth factor D (PDGFD), tv 2.	Homo sapiens
NM_033169.2	beta-1,3-N-acetylgalactosaminyltransferase 1 (globoside blood group) (B3GALNT1), tv 4.	Homo sapiens
NM_033212.2	coiled-coil domain containing 102A (CCDC102A).	Homo sapiens
NM_033221.1	tripartite motif-containing 14 (TRIM14), tv 4.	Homo sapiens
NM_033280.2	SEC11 homolog C (S. cerevisiae) (SEC11C).	Homo sapiens
NM_033364.3	chromosome 3 open reading frame 15 (C3orf15).	Homo sapiens
NM_033527.1	cell division cycle 2-like 2 (PITSLRE proteins) (CDC2L2), tv 2.	Homo sapiens
NM_033631.2	leucine zipper protein 1 (LUZP1).	Homo sapiens
NM_052888.2	leucine rich repeat containing 37B (LRRC37B).	Homo sapiens
NM_052900.2	CUB and Sushi multiple domains 3 (CSMD3), tv c.	Homo sapiens
NM_053035.1	mitochondrial ribosomal protein S33 (MRPS33), nuclear gene encoding mitochondrial protein, tv 2	Homo sapiens
NM_053274.2	glomulin, FKBP associated protein (GLMN).	Homo sapiens
NM_053280.3	outer dense fiber of sperm tails 3 (ODF3).	Homo sapiens
NM_053346.1	neuritin (Nrn1).	Rattus norvegicus
NM_053730.1	stromal antigen 3 (Stag3).	Rattus norvegicus
NM_053832.1	forkhead box J1 (Foxj1).	Rattus norvegicus
NM_057094.1	crystallin, beta A2 (CRYBA2), tv 3.	Homo sapiens
NM_057157.1	cytochrome P450, family 26, subfamily A, polypeptide 1 (CYP26A1), tv 2.	Homo sapiens
NM_057195.1	smu-1 suppressor of mec-8 and unc-52 homolog (C. elegans) (Smu1).	Rattus norvegicus
NM_058181.1	chromosome 21 open reading frame 57 (C21orf57), tv 1.	Homo sapiens
NM_078471.3	myosin XVIIIA (MYO18A), tv 1.	Homo sapiens
NM_078474.2	TM2 domain containing 3 (TM2D3), tv 1.	Homo sapiens
NM_080590.1	calcyphosine (CAPS), tv 2.	Homo sapiens
NM_080655.1	chromosome 9 open reading frame 30 (C9orf30).	Homo sapiens
NM_080865.3	G protein-coupled receptor 62 (GPR62).	Homo sapiens
NM_130476.1	MAP-kinase activating death domain (MADD), tv 8.	Homo sapiens
NM_130791.1	WW domain containing oxidoreductase (WWOX), tv 2.	Homo sapiens
NM_130825.1	comparative gene identification transcript 94 (Cgi94).	Rattus norvegicus
NM_133328.2	death effector domain containing 2 (DEDD2).	Homo sapiens
NM_133344.1	RAD17 homolog (S. pombe) (RAD17), tv 7.	Homo sapiens
NM_133362.2	erythroid differentiation regulator 1 (Erdr1).	Mus musculus
NM_133622.1	common salivary protein 1 (LOC171161).	Rattus norvegicus
NM_133761.3	decapping enzyme (Dcp1a).	Mus musculus
NM_134387.1	dicarbonyl L-xylulose reductase (Dcxr).	Rattus norvegicus
NM_138432.2	serine dehydratase-like (SDSL).	Homo sapiens
NM_138691.2	transmembrane channel-like 1 (TMC1).	Homo sapiens
NM_138698.2	prematurely terminated mRNA decay factor-like (LOC91431).	Homo sapiens

Supplemental Table 1: Negative set of Mammalian AUG, non-aTIS, mRNA Sequences

Accession	Protein	Species
NM_138727.2	suppression of tumorigenicity 7 like (ST7L), tv 2.	Homo sapiens
NM_138789.3	PIH1 domain containing 2 (PIH1D2), tv 1.	Homo sapiens
NM_138822.1	peptidylglycine alpha-amidating monooxygenase (PAM), tv 4.	Homo sapiens
NM_139004.2	hemochromatosis (HFE), tv 4.	Homo sapiens
NM_144597.1	chromosome 15 open reading frame 40 (C15orf40).	Homo sapiens
NM_144751.1	resection-induced TPI (rs11) (LOC246267).	Rattus norvegicus
NM_144769.2	forkhead box I1 (FOXI1), tv 2.	Homo sapiens
NM_144781.1	programmed cell death 2 (PDCD2), tv 2.	Homo sapiens
NM_145060.2	chromosome 18 open reading frame 24 (C18orf24), tv 2.	Homo sapiens
NM_145102.2	zinc finger with KRAB and SCAN domains 5 (ZKSCAN5), tv 2.	Homo sapiens
NM_145129.2	cholinergic receptor, nicotinic, alpha polypeptide 3 (Chrna3).	Mus musculus
NM_145350.1	scavenger receptor class F, member 1 (SCARF1), tv 3.	Homo sapiens
NM_145473.2	cold shock domain containing C2, RNA binding (Csdc2).	Mus musculus
NM_145754.2	kinesin family member C2 (KIFC2).	Homo sapiens
NM_145812.1	apoptosis-inducing factor, mitochondrion-associated, 1 (AIFM1), tv 2.	Homo sapiens
NM_145878.1	fatty acid binding protein 5, epidermal (Fabp5).	Rattus norvegicus
NM_147136.1	rRNA promoter binding protein (LOC257642).	Rattus norvegicus
NM_147138.2	ZW10 interactor (Zwint).	Rattus norvegicus
NM_147177.1	RuvB-like protein 1 (Ruvbl1).	Rattus norvegicus
NM_148894.2	family with sequence similarity 44, member A (FAM44A).	Homo sapiens
NM_152279.2	zinc finger protein 585B (ZNF585B).	Homo sapiens
NM_152280.2	synaptotagmin XI (SYT11).	Homo sapiens
NM_152288.1	transmembrane protein 142C (TMEM142C).	Homo sapiens
NM_152348.2	WD repeat domain 81 (WDR81).	Homo sapiens
NM_152352.2	chromosome 18 open reading frame 19 (C18orf19).	Homo sapiens
NM_152394.3	chromosome 3 open reading frame 44 (C3orf44).	Homo sapiens
NM_152457.1	zinc finger protein 597 (ZNF597).	Homo sapiens
NM_152485.2	chromosome 1 open reading frame 74 (C1orf74).	Homo sapiens
NM_152626.2	zinc finger protein 92 (ZNF92), tv 2.	Homo sapiens
NM_152735.3	zinc finger and BTB domain containing 9 (ZBTB9).	Homo sapiens
NM_153283.1	hyaluronoglucosaminidase 1 (HYAL1), tv 3.	Homo sapiens
NM_153714.1	chromosome 10 open reading frame 67 (C10orf67).	Homo sapiens
NM_170694.1	serine hydrolase-like (SERHL).	Homo sapiens
NM_172113.1	eyes absent homolog 2 (Drosophila) (EYA2), tv 2.	Homo sapiens
NM_173206.2	protein inhibitor of activated STAT, 2 (PIAS2), tv alpha.	Homo sapiens
NM_173325.1	putative retrovirus-related gag protein (LOC286991).	Rattus norvegicus
NM_173326.1	peptide HP (rs14) (LOC286992).	Rattus norvegicus
NM_173454.1	phosphodiesterase 8A (PDE8A), tv 2.	Homo sapiens
NM_173499.3	spermatogenesis associated 8 (SPATA8).	Homo sapiens
NM_173519.1	hypothetical protein MGC34646 (MGC34646).	Homo sapiens
NM_173556.3	coiled-coil domain containing 83 (CCDC83).	Homo sapiens
NM_173837.2	Bcl-2 binding component 3 (Bbc3).	Rattus norvegicus
NM_174463.2	SNRPN upstream reading frame (SNURF).	Bos taurus
NM_174691.1	ferredoxin reductase (FDXR).	Bos taurus
NM_174768.2	serpin peptidase inhibitor, clade A (alpha-1 antiproteinase, antitrypsin), member 3 (SERPINA3).	Bos taurus
NM_174977.2	SEC14-like 4 (S. cerevisiae) (SEC14L4).	Homo sapiens
NM_175924.2	immunoglobulin-like domain containing receptor 1 (ILDR1).	Homo sapiens
NM_176806.2	molybdenum cofactor synthesis 2 (MOCS2), tv 1.	Homo sapiens
NM_177060.3	RIKEN cDNA 9930039A11 gene (9930039A11Rik).	Mus musculus
NM_177536.1	sulfotransferase family, cytosolic, 1A, phenol-preferring, member 1 (SULT1A1), tv 5.	Homo sapiens
NM_178009.2	diacylglycerol kinase, eta (DGKH), tv 2.	Homo sapiens
NM_178153.1	doublecortex; lissencephaly, X-linked (doublecortin) (DCX), tv 3.	Homo sapiens
NM_178272.1	paired immunoglobin-like type 2 receptor alpha (PILRA), tv 2.	Homo sapiens
NM_178544.2	zinc finger protein 546 (ZNF546).	Homo sapiens
NM_178710.3	SNF1-like kinase 2 (Snf1lk2).	Mus musculus
NM_178876.2	integrator complex subunit 3 (Ints3), tv 2.	Mus musculus
NM_180990.2	ligand-gated ion channel, zinc activated 1 (LGICZ1).	Homo sapiens
NM_181368.3	musculoskeletal, embryonic nuclear protein 1 (Mustn1).	Rattus norvegicus
NM_181553.1	CKLF-like MARVEL transmembrane domain containing 3 (CMTM3), tv 2.	Homo sapiens

Supplemental Table 1: Negative set of Mammalian AUG, non-aTIS, mRNA Sequences

Accession	Protein	Species
NM_182483.1	NSFL1 (p97) cofactor (p47) (NSFL1C), tv 3.	Homo sapiens
NM_182822.2	hypothetical protein LOC313453 (RGD727788).	Rattus norvegicus
NM_182908.3	dehydrogenase/reductase (SDR family) member 2 (DHRS2), tv 1.	Homo sapiens
NM_183231.1	IKAROS family zinc finger 3 (Aiolos) (IKZF3), tv 5.	Homo sapiens
NM_183333.1	keratin complex 2, basic, gene 5 (Krt2-5).	Rattus norvegicus
NM_194261.1	ubiquitin-conjugating enzyme E2I (UBC9 homolog, yeast) (UBE2I), tv 4.	Homo sapiens
NM_194354.2	promethin (LOC378467).	Rattus norvegicus
NM_198212.1	caveolin 2 (CAV2), tv 2.	Homo sapiens
NM_198216.1	small nuclear ribonucleoprotein polypeptides B and B1 (SNRPB), tv 1.	Homo sapiens
NM_198251.2	ring finger protein 133 (Rnf133).	Mus musculus
NM_198321.2	UDP-N-acetyl-alpha-D-galactosamine:polypeptide N-acetylgalactosaminyltransferase 10, tv 1	Homo sapiens
NM_198383.1	calcium channel, voltage-dependent, T type, alpha 1G subunit (CACNA1G), tv 6.	Homo sapiens
NM_198385.1	calcium channel, voltage-dependent, T type, alpha 1G subunit (CACNA1G), tv 4.	Homo sapiens
NM_198465.2	Nik related kinase (NRK).	Homo sapiens
NM_198565.1	leucine rich repeat containing 33 (LRRC33).	Homo sapiens
NM_198682.1	glycophorin E (GYPE), tv 2.	Homo sapiens
NM_198859.2	prickle homolog 2 (Drosophila) (PRICKLE2).	Homo sapiens
NM_198968.2	DAZ interacting protein 1 (DZIP1), tv 2.	Homo sapiens
NM_199334.2	thyroid hormone receptor, alpha (THRA), tv 1.	Homo sapiens
NM_201265.1	ubiquitin-like 7 (bone marrow stromal cell-derived) (UBL7), tv 2.	Homo sapiens
NM_201630.1	leucine rich repeat neuronal 2 (LRRN2), tv 2.	Homo sapiens
NM_202467.1	GIPC PDZ domain containing family, member 1 (GIPC1), tv 2.	Homo sapiens
NM_203350.1	zinc finger, RAN-binding domain containing 2 (ZRANB2), tv 1.	Homo sapiens
NM_203476.1	porcupine homolog (Drosophila) (PORCN), tv E.	Homo sapiens
NM_206818.1	osteoclast-associated receptor (OSCAR), tv 1.	Homo sapiens
NM_206895.1	ASCL830 (UNQ830).	Homo sapiens
NM_206962.1	protein arginine methyltransferase 2 (PRMT2), tv 1.	Homo sapiens
NM_206963.1	retinoic acid receptor responder (tazarotene induced) 1 (RARRES1), tv 1.	Homo sapiens

A set of 500 sequences were selected from Mammalian species that translate with one or more AUG start sites.
These sequences were selected from the validated RefSeq database.
