# Supplementary material for: Bioinformatic analyses of mammalian 5'-UTR sequence properties of mRNAs predicts alternative translation initiation sites
Source: BMC Bioinformatics. 2008 May 8;9:232. doi: 10.1186/1471-2105-9-232 (PMC2396638; doi:10.1186/1471-2105-9-232)
Supplement: Additional file 2 — Provisional aTIS RefSeq mRNA Sequences. The provisional set of sequences were selected both from the annotated provisional non-AUG start sites and conserved non-AUG start sites found in aligned 120 bp fragments containing the alternative start sites from the positive aTIS training set. [file 1471-2105-9-232-S2.pdf]

Supplemental Table 2: Provisional aTIS RefSeq mRNA Sequences

Accession	Protein Name	codon	species
NM_001038632	DNase X (LOC654408)	ctg	Sus scrofa
NM_133190	calcium channel, voltage-dependent, gamma subunit 8 (Cacng8)	ctg	Mus musculus
XM_001090384	PREDICTED: BCL2-associated athanogene	ctg	Macaca mulatta
XM_001116068	PREDICTED: Voltage-dependent calcium channel gamma-8 subunit	ctg	Macaca mulatta
XM_849171	Voltage-dependent calcium channel gamma-8 subunit	tgg	Canis familiaris
L33264	CDC2-related protein kinase (PISSLRE)	ctg	Homo sapiens
XR_020408.1	PREDICTED: PISSLRE (LOC737753)	ctg	Pan troglodytes
U73824.1	p97	gtg	Homo sapiens
U76111	translation repressor NAT1	gtg	Homo sapiens
XM_001170734	PREDICTED: eukaryotic translation initiation factor 4 g2, tv 16 (EIF4	gtg	Pan troglodytes
AY513274.1	aging-associated protein 1 (AAG1)	gtg	Homo sapiens
NM_013507.3	eukaryotic translation initiation factor 4, gamma 2 (Eif4g2), tv 1	gtg	Mus musculus
U76113	Oryctolagus cuniculus translation repressor NAT1 mRNA	gtg	Oryctolagus cuniculus
XM_858867.1	PREDICTED: Eukaryotic translation initiation factor 4 g2 (eIF4G 2)	gtg	Canis familiaris
XM_514571.2	PREDICTED: hemopoietic cell kinase (HCK)	ctg	Pan troglodytes
XM_001166452.1	PREDICTED: JAW1-related protein (MRVI1)	ctg	Pan troglodytes
XM_001099716.1	PREDICTED: JAW1-related protein isoform a, tv 3 (LOC702830)	ctg	Macaca mulatta
XM_001137214.1	PREDICTED: MyoD family inhibitor domain containing isoform	gtg	Pan troglodytes
XM_001164025.1	PREDICTED: pregnane X receptor, tv 2 (NR1I2)	ctg	Pan troglodytes
NM_001018042.1	trans-acting transcription factor 3 (Sp3)	ata	Mus musculus
XM_846960	PREDICTED: trans-acting transcription factor 3 isoform 1	ata	Canis familiaris
XM_001166908.1	PREDICTED: stromal interaction molecule 2	ttg	Pan troglodytes
XM_538879.2	PREDICTED: Transcriptional enhancer factor TEF-5 (TEAD-3)	ata	Canis familiaris
XM_605145.2	PREDICTED: TEA domain family member 4 isoform 1 (LOC526771)	ttg	Bos taurus
XM_001138640.1	PREDICTED: Wilms tumor 1 (WT1)	ctg	Pan troglodytes
XM_001084704.1	PREDICTED: Wilms tumor 1 isoform B (LOC696061)	ctg	Macaca mulatta
XM_001162510.1	PREDICTED: proto-oncogene (JUND)	gag	Pan troglodytes
NM_005354.3	proto-oncogene (JUND)	gag	Homo sapiens
XM_864639.1	PREDICTED: proto-oncogene, tv 2 (LOC517192)	gag	Bos taurus
XM_847192.1	PREDICTED: Transcription factor jun-D, tv 2	gag	Canis familiaris
XM_001075655.1	PREDICTED: leukocyte tyrosine kinase (Ltk), tv 3	ctg	Rattus norvegius
XM_001075610.1	PREDICTED: leukocyte tyrosine kinase (Ltk), tv 1	ctg	Rattus norvegius
NM_001044263.1	regulatory factor X-associated protein	acg	Rattus norvegius
HSU63824	transcription factor RTEF-1 (RTEF1) mRNA, complete cds	ttg	Homo sapiens
AF054589	HIC protein isoform p40 and HIC protein isoform p32	gtg	Homo sapiens
M57423	phosphoribosylpyrophosphate synthetase subunit III mRNA	acg	Homo sapiens
NM_002820	parathyroid hormone-like hormone (PTHLH), tv 2	ctg	Homo sapiens
NM_001032882	pregnane X receptor (PXR)	ctg	Macaca mulatta
NM_138606	proviral integration site 2 (Pim2)	ctg	Mus musculus
NM_001014223	deoxyribonuclease 1-like 1 (Dnase1l1)	ctg	Rattus norvegius
NM_027109	deoxyribonuclease 1-like 1 (Dnase1l1)	ctg	Mus musculus
NM_001099456	neuropeptide W (NPW)	ctg	Homo sapiens
X52621	leukocyte tyrosine kinase (Ltk)	ctg	Mus musculus

The provisional set of sequences were selected both from the annotated provisional non-AUG start sites and conserved non-AUG start sites found in aligned 120bp fragments containing the alternative start sites from the positive aTIS training set.
